# An Alternative and Conserved Cell Wall Enzyme That Can Substitute for the Lipid II Synthase MurG

**DOI:** 10.1128/mBio.03381-20

**Published:** 2021-04-06

**Authors:** L. Zhang, K. Ramijan, V. J. Carrión, L. T. van der Aart, J. Willemse, G. P. van Wezel, D. Claessen

**Affiliations:** a Molecular Biotechnology, Institute of Biology, Leiden University, Leiden, The Netherlands; Max Planck Institute for Terrestrial Microbiology

**Keywords:** peptidoglycan, MurG, L-form, morphology switch, cell wall biosynthesis

## Abstract

The cell wall is a stress-bearing structure and a unifying trait in bacteria. Without exception, synthesis of the cell wall involves formation of the precursor molecule lipid II by the activity of the essential biosynthetic enzyme MurG, which is encoded in the division and cell wall synthesis (*dcw*) gene cluster. Here, we present the discovery of a cell wall enzyme that can substitute for MurG. A mutant of Kitasatospora viridifaciens lacking a significant part of the *dcw* cluster, including *murG*, surprisingly produced lipid II and wild-type peptidoglycan. Genomic analysis identified a distant *murG* homologue, which encodes a putative enzyme that shares only around 31% amino acid sequence identity with MurG. We show that this enzyme can replace the canonical MurG, and we therefore designated it MglA. Orthologues of *mglA* are present in 38% of all genomes of *Kitasatospora* and members of the sister genus *Streptomyces*. CRISPR interference experiments showed that *K. viridifaciens mglA* can also functionally replace *murG* in Streptomyces coelicolor, thus validating its bioactivity and demonstrating that it is active in multiple genera. All together, these results identify MglA as a bona fide lipid II synthase, thus demonstrating plasticity in cell wall synthesis.

## Abstract (precis)

Almost all bacteria are surrounded by a cell wall, which protects cells from environmental harm. Formation of the cell wall requires the precursor molecule lipid II, which in bacteria is universally synthesized by the conserved and essential lipid II synthase MurG.

## INTRODUCTION

Bacteria are surrounded by a cell wall, which is a highly dynamic structure that provides cellular protection and dictates cell shape. A major component of the cell wall is peptidoglycan (PG), which is widely conserved in the bacterial domain. Its biosynthesis has been studied for many decades, reinforced by the notion that many successful antibiotics target important steps in this pathway. The first steps of the PG synthesis pathway occur in the cytoplasm, where the peptidoglycan precursor UDP-MurNAc-pentapeptide is synthesized by the consecutive activities of a number of so-called Mur enzymes (MurA to -F) (1). Next, this pentapeptide precursor is linked to undecaprenyl phosphate (or bactoprenol) residing in the plasma membrane by MurX (or MraY), yielding lipid I. UDP-*N*-acetylglucosamine–*N*-acetylmuramyl-(pentapeptide) pyrophosphoryl–undecaprenol *N*-acetylglucosamine transferase (MurG) then adds the sugar nucleotide UDP-GlcNAc to lipid I to form lipid II, which is the complete PG subunit that is flipped to the external side of the membrane. Among the candidates to mediate this flipping *in vivo*, MurJ and AmJ have been proposed, while FtsW was suggested based on *in vitro* analyses (2–4). Following flipping to the exterior of the cell, the PG subunit is then used to synthesize glycan strands by the activities of transglycosylases, after which these strands are cross-linked using transpeptidases (5–8). Many of the genes required for the biosynthesis of PG and for cell division are located in the so-called *dcw* gene cluster (for division and cell wall synthesis) (9, 10) (see Fig. S1 in the supplemental material). The content and organization of the *dcw* cluster are generally conserved among species with similar morphologies, indicating a putative role in bacterial cell shape (11).

10.1128/mBio.03381-20.1 FIG S1 Morphology of *Kitasatospora viridifaciens* with and without the cell wall. Transmission electron microscopy of a wall-deficient cell of *alpha* (A) and the parental wild-type strain DSM40239 (B). The arrowheads in panel B indicate the cell wall. Scale bars, 500 nm (A) and 250 nm (B). Download FIG S1, TIF file, 1.0 MB. Copyright © 2021 Zhang et al. 2021 Zhang et al. https://creativecommons.org/licenses/by/4.0/ This content is distributed under the terms of the Creative Commons Attribution 4.0 International license.

Members of the *Streptomycetaceae* within the actinobacteria are filamentous Gram-positive soil bacteria that have a complex multicellular life cycle (12, 13). The best-studied genus is *Streptomyces*, which is industrially highly relevant, as it produces over half of all known antibiotics used in clinics and many other bioactive compounds with clinical or agricultural application (14, 15). The life cycle of streptomycetes starts with the germination of a spore, and the arising vegetative hyphae grow out via tip extension and branching to form a dense network called the vegetative mycelium. The vegetative mycelium consists of long multinucleated syncytial cells separated by widely spaced cross walls (16, 17). The reproductive phase is initiated by the formation of an aerial mycelium, by which the vegetative hyphae are cannibalized as a substrate (18, 19). The aerial hyphae then differentiate into chains of unigenomic spores. During sporulation, the conserved cell division protein FtsZ assembles as foci along the hyphal wall, eventually resulting in a ladder of regularly spaced Z-rings that form the cell division scaffold (20). This is followed by a process of cytokinesis, which results in spore formation, following a complex process of coordinated cell division and DNA segregation (21, 22).

Comparison between *Bacillus* and *Streptomyces* shows that some cell division-related proteins have evolved different functionalities between firmicutes and actinobacteria. An example of such a divergent function is exemplified by DivIVA; in Bacillus subtilis, this protein is involved in selection of the division site by preventing polar accumulation of FtsZ (23), while DivIVA in actinobacteria plays an essential role in polar growth (24). Thus, *divIVA* cannot be deleted in actinobacteria, while it is dispensable in B. subtilis. Conversely, many cell division genes, including *ftsZ*, can be deleted in actinobacteria, while they are essential for unicellular microbes. This makes actinobacteria intriguing model systems for the study of cell division and growth (21, 25). It is also worth noticing that the streptomycetes have a complex cytoskeleton, with many intermediate filament-like proteins required for hyphal integrity (26–29).

Besides the genus *Streptomyces*, the family *Streptomycetaceae* also encompasses the genera *Kitasatospora* and *Streptacidiphilus*. While these genera are highly similar in growth and development, *Kitasatospora* is distinct from *Streptomyces* (30, 31). For instance, the compositions of the cell wall are different between members of these genera, and several regulatory proteins required for morphogenesis in *Streptomyces* are absent in *Kitasatospora* (31). We recently described that *Kitasatospora viridifaciens* releases cell wall-deficient cells, called S-cells, under conditions of hyperosmotic stress (32). These S-cells are only transiently wall deficient and can switch to the mycelial mode of growth. In some cases, however, prolonged exposure to high levels of osmolytes can lead to the emergence of mutants that are able to proliferate in the wall-deficient state as so-called L-forms (32, 33). Like S-cells, these L-forms retain the ability to construct functional peptidoglycan based on the observation that removal of the osmolytes from the medium leads to the formation of mycelial colonies. L-forms can also be generated in most other bacteria by exposing cells to compounds that target the process of cell wall synthesis (33–35). Strikingly, such wall-deficient cells can propagate without the FtsZ-based cell division machinery (35–37). Even though the procedures used to generate L-forms can markedly differ, their mode of proliferation is conserved across species and largely based on biophysical principles. An imbalance in the cell surface area/volume ratio in cells that increase in size causes strong deformations of the cell membrane, followed by the release of progeny cells by blebbing, tubulation, and vesiculation (32, 38). Given that lipid vesicles without any content can proliferate in a manner similar to that observed for L-forms led to the hypothesis that this mode of proliferation may be comparable to that used by early life forms that existed before the cell wall had evolved (39, 40).

Here, we exploited the unique properties of a *K. viridifaciens* L-form strain that readily switches between a wall-deficient and filamentous mode of growth to discover an alternative MurG-like enzyme that is important for building the PG-based cell wall. Our data surprisingly show that *K. viridifaciens* produces wild-type peptidoglycan in the absence of *murG*, which has so far been considered essential for lipid II biosynthesis in all bacteria. The MurG activity is taken over by a homologue called MglA, which is widespread in filamentous actinobacteria and able to substitute for the absence of MurG across different genera.

## RESULTS

### Morphological transitions of the shape-shifting strain *alpha*.

**FIG 1:**
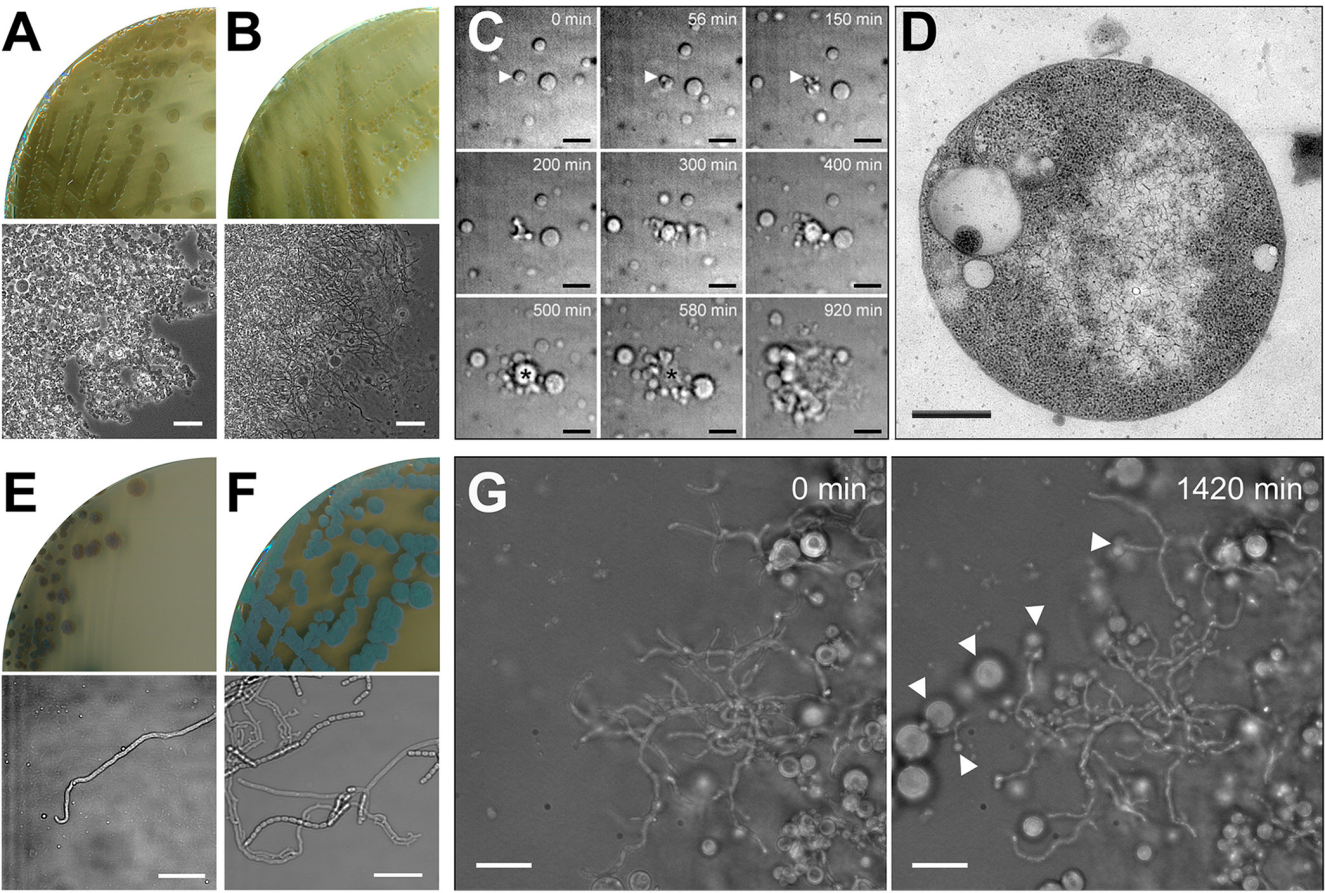
Morphological transitions of the shape-shifting strain *alpha.* (A) Growth of the *K. viridifaciens alpha* strain on LPMA medium yields greenish, mucoid colonies exclusively consisting of L-form cells, unlike the wild-type strain that forms yellowish colonies consisting of mycelia and S-cells (B). (C) Time-lapse microscopy stills of *alpha* proliferating in the wall-deficient state in liquid LPB medium. The arrowhead shows the mother cell, which generates progeny and lyses after 580 min (marked with an asterisk). Stills were taken from Movie S1A in the supplemental material. (D) Transmission electron microscopy of a wall-deficient cell of *alpha.* (E) Growth of *alpha* on solid MYM yields compact, nonsporulating colonies unlike those of the wild-type strain, which forms gray-pigmented sporulating colonies (F). (G) Time-lapse microscopy stills of the mycelium of *alpha* transferred to LPMA medium, showing the extrusion of L-forms by filaments (arrowheads). Stills were taken from Movie S1B. Scale bars represents 20 μm (A, B), 10 μm (C, E, F, G), and 500 nm (D).

We recently generated a K. viridifaciens L-form lineage by exposing the parental wild-type strain to high levels of penicillin and lysozyme. This strain, designated *alpha*, proliferates indefinitely in the cell wall-deficient state in media containing high levels of osmolytes (32). On solid L-phase medium (LPMA), containing high levels of sucrose, *alpha* forms greenish viscous colonies, which exclusively contain L-form cells (Fig. 1A). In contrast, the parental strain forms compact, yellowish colonies composed of mycelia and S-cells on LPMA medium (Fig. 1B). Likewise, in liquid Baird-Parker liquid (LPB) medium, which like LPMA medium contains high levels of sucrose, *alpha* proliferates exclusively in the wall-deficient state in a manner that is morphologically similar to that described for other L-forms (35, 41, 42) (see Movie S1A in the supplemental material; Fig. 1C). Following strong deformations of the mother cell membrane (see panels for 56, 150, and 200 min in Fig. 1C), small progeny cells are released after approximately 300 min. The mother cell, from which the progeny were released (indicated with an asterisk in Fig. 1C), lysed after 580 min. Characterization using transmission electron microscopy (TEM) confirmed that *alpha* possessed no PG-based cell wall when grown on media containing high levels of osmolytes (Fig. 1D; Fig. S1). Notably, when *alpha* is plated on maltose-yeast extract medium (MYM; lacking high levels of osmolytes) the strain can switch to the mycelial mode of growth (Fig. 1E). However, unlike the wild-type strain (Fig. 1F), the mycelial colonies of *alpha* fail to develop aerial hyphae and spores. Subsequent transfer of mycelia to LPMA medium plates stopped filamentous growth and reinitiated wall-deficient growth, during which L-form cells were extruded from stalled hyphal tips (Movie S1B; Fig. 1G). Given the ability of these wall-deficient cells to proliferate, they eventually dominated the culture (not shown). Taken together, these results demonstrate that *alpha* can switch between a walled and wall-deficient state.

10.1128/mBio.03381-20.9 MOVIE S1A L-form proliferation of *alpha.* Time-lapse microscopy showing proliferation of *alpha* in LPB medium containing high levels of sucrose. The times are indicated in minutes. Scale bar, 5 μm. Download Movie S1, AVI file, 1.4 MB. Copyright © 2021 Zhang et al. 2021 Zhang et al. https://creativecommons.org/licenses/by/4.0/ This content is distributed under the terms of the Creative Commons Attribution 4.0 International license.

10.1128/mBio.03381-20.10 MOVIE S1B Extrusion of L-forms from hyphal tips in *alpha*. Cell wall-deficient L-forms are extruded from hyphal tips when mycelium of *alpha* is transferred to LPMA agar containing high levels of sucrose. The times are indicated in minutes. Scale bar, 5 μm. Download Movie S1, AVI file, 14.8 MB. Copyright © 2021 Zhang et al. 2021 Zhang et al. https://creativecommons.org/licenses/by/4.0/ This content is distributed under the terms of the Creative Commons Attribution 4.0 International license.

### Deletion of *divIVA* abolishes switching of *alpha* from the wall-deficient to the filamentous mode of growth.

**FIG 2:**
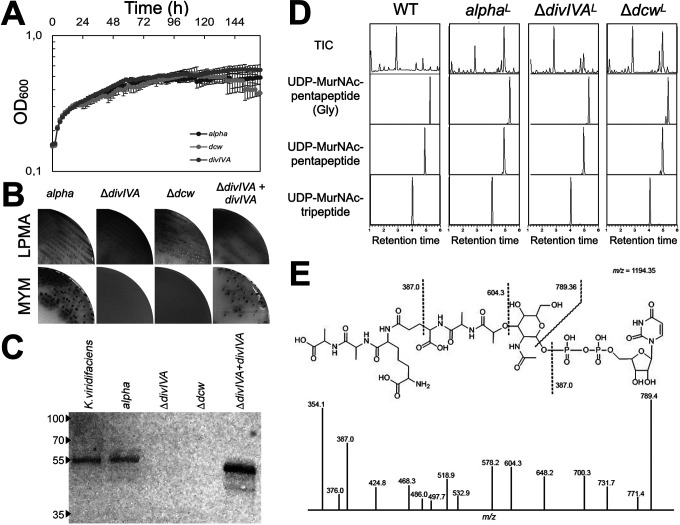
The absence of DivIVA abolishes switching of *alpha* from the wall-deficient to the filamentous mode of growth. (A) Growth curves of *alpha* (black spheres), the *divIVA* mutant (gray squares), and the *dcw* mutant (gray triangles) in liquid LPB medium. All values represent the averages and standard deviations of results from three replicates. (B) While all strains grow on LPMA medium, those lacking *divIVA* are unable to switch to the mycelial mode of growth on MYM lacking osmoprotectants. (C) Western blot analysis using antibodies against the C. glutamicum DivIVA protein confirm the absence of DivIVA in the constructed Δ*divIVA* and *dcw* mutants. Reintroduction of *divIVA* under the control of the strong, nonnative *gap1* promoter restores the expression of DivIVA in the *divIVA* mutant and the ability to form mycelial colonies (B). Numbers on the left are molecular masses (in kilodaltons). (D) Qualitative LC-MS analysis of peptidoglycan precursors in *alpha* and its *divIVA* and *dcw* mutants. Three cytoplasmic PG precursors are shown: undecaprenyl-*N*-acetylmuramic acid–l-Ala–d-Glu–l,l-meso-diaminopimelic acid (DAP) (UDP-MurNAc-tripeptide), UDP–MurNAc–l-Ala–d-Glu–l,l-*meso*-DAP–d-Ala–d-Ala (UDP-MurNAc-pentapeptide), and a pentapeptide with DAP-bound Gly (UDP-MurNAc-pentapeptide Gly). Like the wild-type, all strains produce these peptidoglycan precursors, including UDP-MurNAc-pentapeptide, which is the last cytosolic precursor in the PG biosynthesis pathway. TIC, total ion current. Superscript L denotes L-form. (E) MS-MS analysis confirming that the product with a mass of 1,194.35 is the precursor UDP-MurNAc-pentapeptide.

The ability of *alpha* to efficiently switch between the walled and wall-deficient state provides an ideal platform to delete genes essential for cell wall biosynthesis. As a proof of concept, we focused on *divIVA*, which is essential for polar growth in filamentous actinomycetes (24). In actinobacteria, *divIVA* is located adjacent to the conserved *dcw* gene cluster (Fig. S2). *divIVA* is present in Gram-positive rod-shaped (*Mycobacterium*, *Corynebacterium*, *Bacillus*), filamentous (*Streptomyces* and *Kitasatospora*), and coccoid (*Staphylococcus* and *Streptococcus*) bacteria but absent in Gram-negative bacteria, such as Escherichia coli. In B. subtilis and Staphylococcus aureus, the DivIVA proteins share only 29% (BSU15420) and 26% (SAOUHSC_01158) amino acid identity to the Streptomyces coelicolor orthologue. To localize DivIVA, plasmid pKR2 was created, allowing constitutive expression of DivIVA-enhanced green fluorescent protein (eGFP) (see Materials and Methods). Fluorescence microscopy revealed that the fusion protein localized to hyphal tips (Fig. S3A), as in streptomycetes (24). When *alpha* was grown in the wall-deficient state in LPB medium, typically one or two foci of DivIVA-eGFP were detected per cell, which invariably were localized to the membrane. In contrast, no foci were detected in L-form cells containing the empty plasmid (pKR1) or those expressing cytosolic eGFP (pGreen [43]). We then constructed the plasmids pKR3 to delete *divIVA* and pKR4 to delete a large part of the *dcw* gene cluster, including *divIVA* (see Materials and Methods). Introduction of these plasmids into *alpha* by polyethylene glycol (PEG)-mediated transformation and a subsequent screening yielded the desired *divIVA* and *dcw* mutants (Fig. S4). Analysis of growth in LPB medium or on solid LPMA plates indicated that the L-form cells proliferated normally in the absence of *divIVA* or part of the *dcw* gene cluster (Fig. 2A). However, when L-form cells were plated on MYM (lacking osmoprotectants), only the *alpha* strain was able to switch to the mycelial mode of growth (Fig. 2B). Introduction of plasmid pKR6, which expresses *divIVA* from the constitutive *gap1* promoter, complemented the growth of the *divIVA* mutant on MYM (Fig. 2B). In agreement, Western blot analysis using antibodies against DivIVA of Corynebacterium glutamicum confirmed the absence of DivIVA in both the *divIVA* and the *dcw* mutant and showed that expression was restored in the *divIVA* mutant complemented with pKR6 (Fig. 2C).

10.1128/mBio.03381-20.2 FIG S2 Comparative analysis of *dcw* gene clusters from different bacteria. (A) Organization and content of the *dcw* gene cluster from Streptomyces coelicolor A3(2). (B) MultiGeneBlast output showing homologous *dcw* gene clusters with a minimal identity of 30% and minimal sequence coverage of 25% to the S. coelicolor cluster. Download FIG S2, TIF file, 1.1 MB. Copyright © 2021 Zhang et al. 2021 Zhang et al. https://creativecommons.org/licenses/by/4.0/ This content is distributed under the terms of the Creative Commons Attribution 4.0 International license.

10.1128/mBio.03381-20.3 FIG S3 Localization of DivIVA-eGFP in *alpha.* (A) Fluorescence microscopy analysis of *alpha* grown in TSBS medium as a mycelium and carrying pKR1 (left panels), pGreen (middle panels), or pKR2 (right panels). In mycelium containing pKR2, DivIVA-eGFP is localized at the hyphal tips (see the arrowheads in the right panels). No fluorescence is observed in mycelium containing the control plasmid pKR1 (left panels), while a cytosolic signal is observed in *alpha* transformed with pGreen (middle panels). (B) Fluorescence microscopy analysis of *alpha* grown in LPB medium in the wall-deficient state and carrying pKR1 (left panels), pGreen (middle panels), and pKR2 (right panels). Cells expressing the DivIVA-eGFP fusion protein show distinct foci localized to the membrane (right panels). As in mycelia, no fluorescence is observed in cells containing the control plasmid pKR1 (left panels), while a cytosolic signal is evident in cells containing pGreen (middle panels). Scale bars, 10 μm. Download FIG S3, TIF file, 2.8 MB. Copyright © 2021 Zhang et al. 2021 Zhang et al. https://creativecommons.org/licenses/by/4.0/ This content is distributed under the terms of the Creative Commons Attribution 4.0 International license.

10.1128/mBio.03381-20.4 FIG S4 PCR verification demonstrating the deletions of *divIVA* and the partial *dcw* gene cluster in *alpha*. (A) Schematic illustration of the *dcw* clusters in *alpha* (top) and the derivative strains lacking *divIVA* (middle) or part of the *dcw* cluster (bottom). To verify the deletions, PCR analyses were performed using primers *divIVA-Fw* and *divIVA-Rv* (B) and *dcw-Fw* and *dcw-Rv* (C). (B) PCR analysis using primers *divIVA-Fw* and *divIVA-Rv* yielded PCR products of 1.8 kb when chromosomal DNA of the wild-type strain (DSM40239) or *alpha* was used, while a 2.7-kb fragment was obtained in the Δ*divIVA* mutant. As expected, no product was obtained with these primers using chromosomal DNA of the *dcw* mutant as the template. (C) PCR analysis using primers *dcw-Fw* and *dcw-Rv* yielded a PCR product of only 1.7 kb when chromosomal DNA of the *dcw* mutant was used as the template. Please note that the sizes of the fragments expected for the wild-type strain and *alpha* (8.2 kb) and the Δ*divIVA* mutant (9.2 kb) are too large for efficient amplification. Download FIG S4, TIF file, 1.2 MB. Copyright © 2021 Zhang et al. 2021 Zhang et al. https://creativecommons.org/licenses/by/4.0/ This content is distributed under the terms of the Creative Commons Attribution 4.0 International license.

To analyze if the switch from the wall-deficient to the walled state in the absence of DivIVA was blocked due to the failure to produce the cytosolic precursors required for peptidoglycan synthesis in the L-form state, we performed a comparative liquid chromatography-mass spectrometry (LC-MS) analysis (Fig. 2D). We noticed that the LC-MS profiles of the *divIVA* and *dcw* mutant strains were similar to that of *alpha* with respect to the cytosolic PG building blocks (Fig. 2D). Importantly, MS-MS analysis identified the last cytosolic precursor in the PG biosynthesis pathway, UDP-MurNAc-pentapeptide (*M*_w_ = 1,194.35) in all strains (Fig. 2E). Taken together, these results demonstrate that DivIVA is essential for filamentous growth but not required for synthesis of the cytosolic PG precursors.

### Identification of a distant MurG homologue as an alternative lipid II synthase.

**FIG 3:**
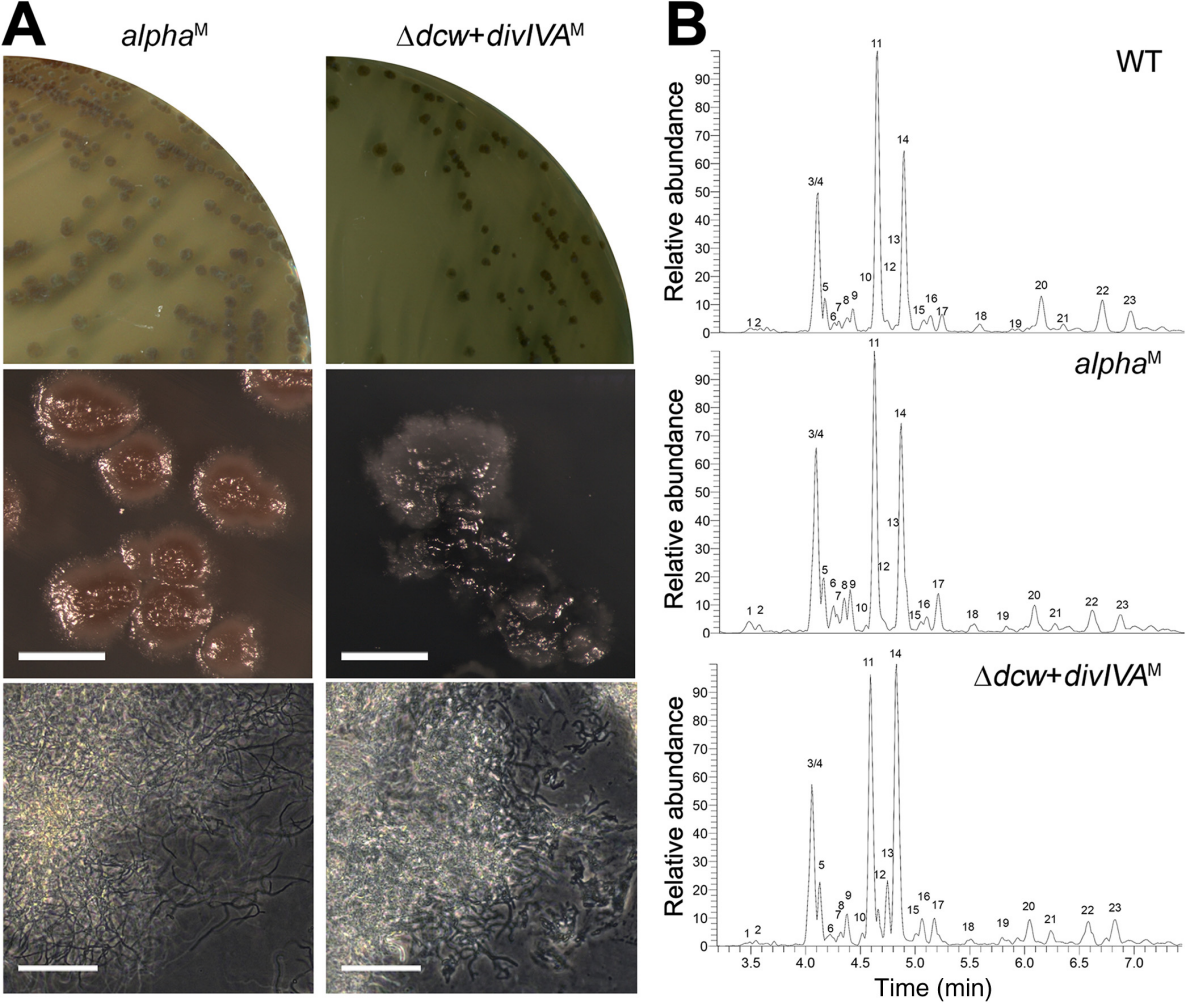
The reintroduction of *divIVA* alone is sufficient to restore the filamentous growth of the *dcw* mutant. (A) Morphological comparison between *alpha* (left) and the *dcw* mutant transformed with P*_gap1_-divIVA* (right) grown on MYM. Unlike *alpha*, the *dcw* mutant expressing DivIVA forms colonies with a heterogenous appearance. Superscript M denotes mycelium. (B) Peptidoglycan architecture analysis of the mycelia of the wild-type strain (top), *alpha* (middle), and the *dcw* mutant expressing DivIVA (bottom). The muropeptide pattern is comparable in all strains despite the lack of *murG* in the *dcw* mutant (see also Table 1). Scale bar, 40 μm.

Having a mutant lacking many genes of the *dcw* cluster offers many opportunities for the study of individual genes. The constructed *dcw* mutant lacks *ftsW*, *murG*, *ftsQ*, *ftsZ*, *ylmD*, *ylmE*, *selF*, *sepG*, and *divIVA*. Surprisingly, introduction of only *divIVA* (expressed from the constitutive *gap1* promoter) (Fig. S5) restored the ability of the *dcw* mutant to switch to the walled mode of growth on solid media lacking osmoprotectants (Fig. 3A). The colonies that were formed were small and heterogeneous compared to the mycelial colonies formed by *alpha* (Fig. 3A). Furthermore, expression of *divIVA* in the *dcw* mutant was not able to restore filamentous growth in liquid cultures (data not shown). To verify that the *dcw* mutant expressing *divIVA* produced normal PG on solid medium, we performed a peptidoglycan architecture analysis using LC-MS (Fig. 3B). This surprisingly revealed that all expected muropeptides were formed at levels comparable to those formed by *alpha* and the wild-type strain, despite the absence of a functional *murG* gene (Fig. 3B; Table 1).

**TABLE 1:** Muropeptides identified in *K. viridifaciens* strains grown as mycelium^*a*^

Peak	Muropeptide	Retention time (min)	Observed mass [M + H]	Calculated mass	% in:		
					Wild type	*alpha*	Δ*dcw divIVA* strain
1	Tri (–Gly)	3.46	870.39	869.38	0.69	1.95	0.48
2	Di [deAc]	3.54	656.30	655.29	0.48	0.10	0.59
3	Di	4.07	698.31	697.30	9.39	10.74	6.55
4	Tri	4.07	927.41	926.41	15.76	22.06	17.34
5	Tetra [Gly4]	4.13	984.44	983.43	3.03	5.16	5.45
6	TriTri (–GM)	4.23	1,355.61	1,354.60	1.16	1.67	0.47
7	Tetra (–Gly)	4.27	941.43	940.42	1.00	1.71	0.67
8	Tri [Glu]	4.34	928.40	927.39	1.59	0.42	1.57
9	Penta [Gly5]	4.38	1,055.47	1,054.47	21.87	4.02	2.98
10	TetraTetra (–GM) [Gly4]	4.52	1,483.67	1,462.66	1.32	2.47	3.45
11	Tetra	4.58	998.45	997.44	26.66	27.63	25.82
12	TetraTri (–GM)	4.66	1,426.65	1,425.64	14.12	18.68	19.13
13	Unidentified peptide	4.75	1,055.50	1,054.47	0.00	0.00	5.76
14	Penta	4.81	1,069.49	1,068.48	17.49	21.81	29.76
15	TetraTri (–GM) [deAc/Gly4]	5.01	1,369.63	1,368.62	6.09	5.96	5.99
16	TetraTetra (–GM)	5.06	1,497.39	1,496.38	6.41	6.35	9.82
17	Penta [Glu]	5.17	1,070.47	1,069.47	2.05	4.40	3.03
18	TriTri	5.52	1,835.81	1,834.81	5.12	5.59	3.75
19	TetraTri [Glu]	6.11	1,906.84	1,905.84	4.60	7.42	2.59
20	TetraTri	6.34	1,907.83	1,906.83	24.69	20.24	17.17
21	TetraTetra [Glu]	6.45	1,977.87	1,976.88	3.97	5.19	7.51
22	TetraTetra	6.67	1,978.88	1,977.86	20.50	15.85	15.20
23	PentaTetra [Glu]	6.94	2,049.91	2,048.90	12.03	10.57	14.93

a Monomers and dimers are treated as separate sets. The mature peptidoglycan is GlcNAc–MurNAc–l-Ala–d-Gln–l,l-*meso*-DAP(Gly)–d-Ala–d-Ala unless DAP-linked Gly is lost (–Gly), MurNAc is deacetylated [deAc] to MurN, there is Gly instead of d-Ala at position 4 [Gly4] or at position 5 [Gly5], there is d-Glu instead of d-Gln [Glu], or dimers can lose one set of GlcNAc-MurNac (–GM). All masses are indicated in daltons.

10.1128/mBio.03381-20.5 FIG S5 Complementation of the *dcw* mutant with *divIVA*. Western blot analysis using antibodies against the C. glutamicum DivIVA protein confirm the absence of DivIVA in the constructed Δ*divIVA* and Δ*dcw* mutants. Reintroduction of *divIVA* under the control of the strong, nonnative *gap1* promoter restores the expression of DivIVA in the *dcw* mutant. Please note that this Western blot also shows a sample of S. coelicolor (whose DivIVA is slightly smaller). Download FIG S5, TIF file, 1.1 MB. Copyright © 2021 Zhang et al. 2021 Zhang et al. https://creativecommons.org/licenses/by/4.0/ This content is distributed under the terms of the Creative Commons Attribution 4.0 International license.

**FIG 4:**
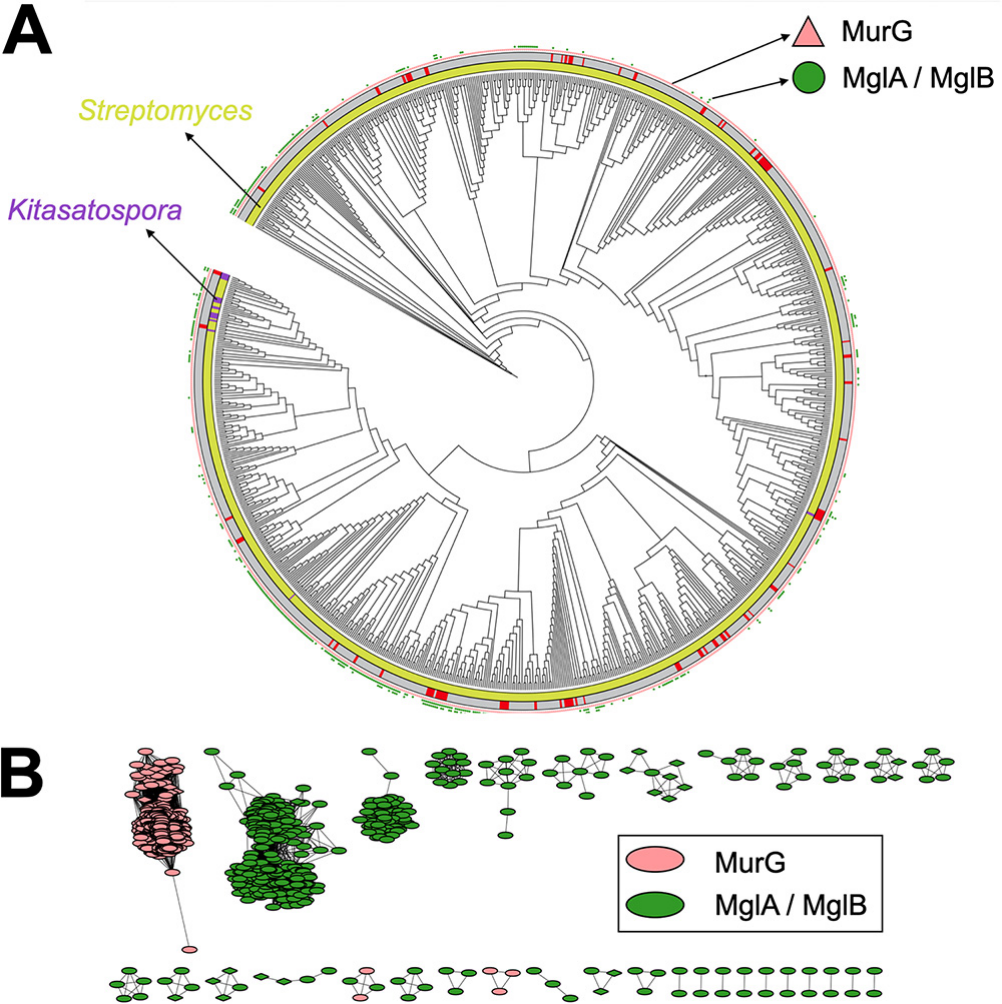
Overview of MurG and MurG-like proteins present in *Streptomyces* and *Kitasatospora* species. (A) The phylogenetic tree was constructed based on four conserved housekeeping proteins (AtpD, RecA, TrpB, and GyrB). Lime green and purple in the inner circle represent *Streptomyces* and *Kitasatospora* species, respectively. Strains present in the NCBI database are indicated in gray in the middle circle, while those from an in-house collection are indicated in red. The pink triangles represent MurG proteins encoded in the *dcw* gene cluster. The green dots represent distant MurG proteins, whose genes are located elsewhere in the genomes. Phylogenetic trees were constructed using iTOL (70). (B) Sequence similarity network of the MurG and MglA/MglB proteins encoded in the genomes of *Streptomyces* and *Kitasatospora* species. Nodes represent MurG proteins, and edges highlight similarity (with a threshold set at 0.9). Node colors indicate if the MurG(-like) proteins are encoded in the *dcw* gene cluster (pink) or elsewhere in the genome (green). Oval-shaped nodes are proteins from *Streptomyces* spp., while those from *Kitasatospora* spp. are shown as diamonds. Please note that almost all MurG proteins encoded in the *dcw* cluster group together (Fig. S7).

The ability of the *dcw* mutant expressing *divIVA* to become filamentous inevitably means that another protein had functionally replaced the activity of MurG. BLAST analysis of the amino acid sequence of MurG from Streptomyces coelicolor (MurG_Sco_, SCO2084) against the genome sequence of *K. viridifaciens* revealed that this actinomycete contains two putative, but distant, MurG homologs (Table 2). The two additional homologs (BOQ63_RS12640 and BOQ63_RS05415) showed 31.2% and 16.5% sequence identity, respectively, to MurG (Fig. S7). Further investigation revealed that MurG proteins possess two characteristic domains: an N-terminal domain that contains the lipid I binding site (PF03033) (44) and a C-terminal domain that contains the UDP-GlcNAc binding site (PF04101) (Fig. S6), both of which are required for the UDP-*N*-acetylglucosamine transferase activity. Of the two distant MurG homologs, only BOQ63_RS12640 contained both domains (Fig. S6). A broader search of MurG-like proteins in other *Streptomyces* and *Kitasatospora* spp. revealed that 38% of the strains possess one, two, and sometimes even three genes for MurG-like proteins containing both the necessary N-terminal (PF03033) and C-terminal (PF04101) domains (Fig. 4A), in addition to canonical MurG, which is present in all strains and encoded in the *dcw* gene cluster. A sequence similarity network was produced by pairwise comparison of the 1,553 MurG and MurG-like proteins extracted from all translated *Streptomyces* and *Kitasatospora* genomes, which showed that nearly all MurG proteins encoded by the orthologue of *murG* in the *dcw* gene cluster grouped together. However, the MurG-like proteins clustered in many different groups (Fig. 4B).

**TABLE 2:** murG homologues in *Kitasatospora viridifaciens*

Hit	Scaffold	Hit start (bp)	Hit end (bp)	Locus	Pairwise identity (%)
1	Chromosome	5334877	5335956	BOQ63_RS32465	100
2	Chromosome	1072546	1073598	BOQ63_RS12640	31.2
3	KVP1 (plasmid)	1258806	1257943	BOQ63_RS05415	16.5

10.1128/mBio.03381-20.6 FIG S6 Domain structures of MurG and MglA proteins. MurG proteins contain an N-terminal domain (PF03033) that binds lipid I and is involved in membrane association. The C-terminal domain (PF04101) contains the UDP-GlcNAc binding site. These domains are found in MurG proteins of E. coli (NCBI accession no. AAC73201.1), B. subtilis (CAB13395.2), S. coelicolor (NP_626343.1), and *K. viridifaciens* (BOQ63_RS32465). Notably, MglA of *K. viridifaciens* (BOQ63_RS12640) also contains both domains. Please note that the protein encoded by the *BOQ63_RS05415* gene contains only the N-terminal domain (PF03033) and not the C-terminal (PF04101) domain. Download FIG S6, TIF file, 1.1 MB. Copyright © 2021 Zhang et al. 2021 Zhang et al. https://creativecommons.org/licenses/by/4.0/ This content is distributed under the terms of the Creative Commons Attribution 4.0 International license.

10.1128/mBio.03381-20.7 FIG S7 Maximum-likelihood tree of MurG homologs in filamentous actinomycetes. The MurG proteins located in the *dcw* gene cluster are grouped and marked with an asterisk (*). The phylogenetic tree was constructed with MEGA5, and the tree reliability was estimated with a bootstrap analysis using 1,000 replicates. Branches with a bootstrap value of less than 50% were collapsed to emphasize reliable branching patterns. The *K. viridifaciens* MurG and MurG homologues (MglA and MglB) are highlighted in bold. Download FIG S7, TIF file, 0.6 MB. Copyright © 2021 Zhang et al. 2021 Zhang et al. https://creativecommons.org/licenses/by/4.0/ This content is distributed under the terms of the Creative Commons Attribution 4.0 International license.

**FIG 5:**
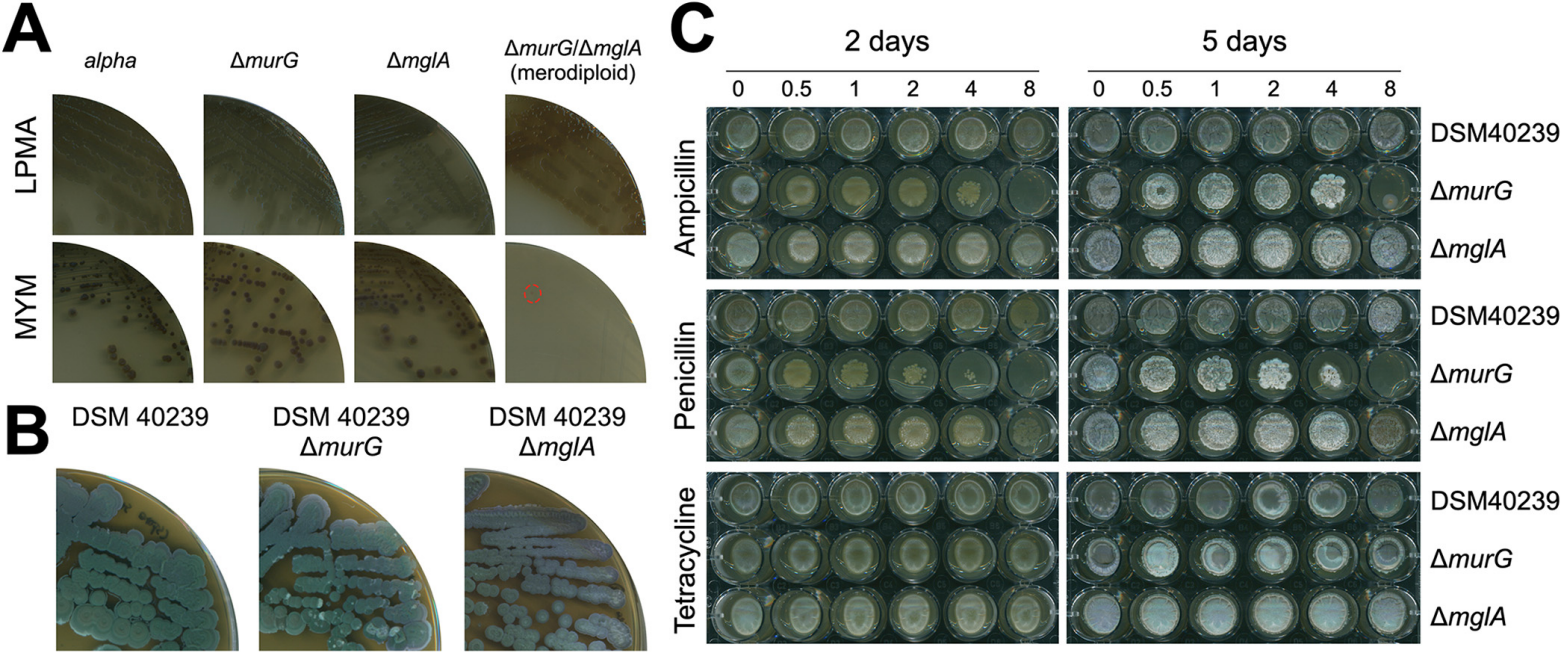
MglA can functionally replace MurG in peptidoglycan synthesis. (A) Growth of *alpha* and the Δ*murG*, Δ*mglA*, and merodiploid Δ*murG* Δ*mglA* mutant strains on LPMA medium (top). Except for the Δ*murG* Δ*mglA* merodiploid, all strains efficiently switched to filamentous growth on MYM lacking osmolytes (bottom). (B) Plates of *K. viridifaciens* and its Δ*murG* and Δ*mglA* mutants grown on MYM for 7 days. (C) Plates of *K. viridifaciens* and the Δ*murG* and Δ*mglA* mutant strains grown on MYM for 2 (left) or 5 (right) days in the presence of ampicillin (top), penicillin (middle), and tetracycline (bottom). The antibiotic concentrations (in micrograms per milliliter) are indicated above the plates.

To corroborate that *murG* is not required for filamentous growth, we decided to delete *murG* in *alpha* using knockout construct pKR8 (see Materials and Methods). The genotype of the mutant was verified by PCR (Fig. S8A) and showed that the absence of *murG* had no effect on L-form or filamentous growth (Fig. 5A). Likewise, inactivation of *mglA* in *alpha* using construct pKR9 had no effect on L-form growth and did not prevent switching to mycelial growth (Fig. 5A). We then attempted to create a double mutant by deleting *mglA* in the *murG* mutant. PCR analysis of a putative double mutant strain with the highly sensitive Q5 DNA polymerase indicated, however, that a small proportion of the multinucleated L-forms had retained a copy of *mglA* (Fig. S8A). Also, further subculturing of this merodiploid strain in the presence of antibiotics that counterselected for maintenance of *mglA* did not lead to a complete loss of this gene, suggesting that the ability to produce lipid II is essential in these L-forms (see Discussion). Nevertheless, plating this merodiploid strain on MYM essentially blocked mycelial growth, and only at very high cell densities were infrequent shifters found (see encircled colony in Fig. 5A).

10.1128/mBio.03381-20.8 FIG S8 PCR analysis demonstrating the *murG* and *mglA* deletions in *alpha* and DSM40239. (A) Verification of the deletions of *murG* and *mglA* in *alpha* using PCR. In strains carrying a wild-type *murG* gene (DSM40239, *alpha* and Δ*mglA*), a fragment of 1,365 bp is amplified. In contrast, a fragment of 1,436 bp is found in the *murG* mutant and the Δ*murG/*Δ*mglA* merodiploid strain (left gel). Likewise, the expected PCR product for strains carrying the *mglA* wild-type gene (DSM40239, *alpha*, Δ*murG*) was 1.2 kb, while replacement of *mglA* by apramycin or viomycin yielded PCR products of 1.3 kb and 1.5 kb, respectively (right gel). Please note that the *mglA* gene is still detectable in the Δ*murG/*Δ*mglA* merodiploid. (B) Verification of the deletions of *murG* and *mglA* in *K. viridifaciens* DSM40239 using PCR. In the wild-type strain (DSM40239) a fragment of 1,365 bp is amplified, while a fragment of 1,436 bp is found in three independent *murG* mutants (Δ*murG*) (left gel). Likewise, the expected size of the PCR product for the wild-type strain carrying the *mglA* gene (DSM40239) was 1,279 bp, while replacement of *mglA* yielded a PCR product of 1,311 bp (Δ*mglA*) (right gel). Download FIG S8, TIF file, 0.7 MB. Copyright © 2021 Zhang et al. 2021 Zhang et al. https://creativecommons.org/licenses/by/4.0/ This content is distributed under the terms of the Creative Commons Attribution 4.0 International license.

Having demonstrated that *murG* is not required for the filamentous growth of *alpha*, we then wondered whether *murG* would also be dispensable for the filamentous growth of the wild-type strain. Notably, *murG* deletion mutants could not be obtained if transformants were selected on MYM, unlike with a *mglA* deletion mutant that was readily found. However, when transformants were selected on LPMA medium containing high levels of sucrose, a *murG* mutant could be created in *K. viridifaciens* (Fig. S8B). As shown in Fig. 5B, the generated *murG* and *mglA* mutants were able to develop and sporulate normally on MYM, compared to the parental wild type. However, exposing the strains to low levels of penicillin and ampicillin revealed that the *murG* mutant was more susceptible to these cell wall-targeting antibiotics than the wild type and its *mglA* mutant. In contrast, no difference effect was observed when tetracycline was added to the plates (Fig. 5C). All together, these results demonstrate that MurG and MglA have overlapping activities, whereby MglA is able to functionally replace the canonical lipid II synthase MurG.

### MglA from *K. viridifaciens* can functionally replace MurG in S. coelicolor.

**FIG 6:**
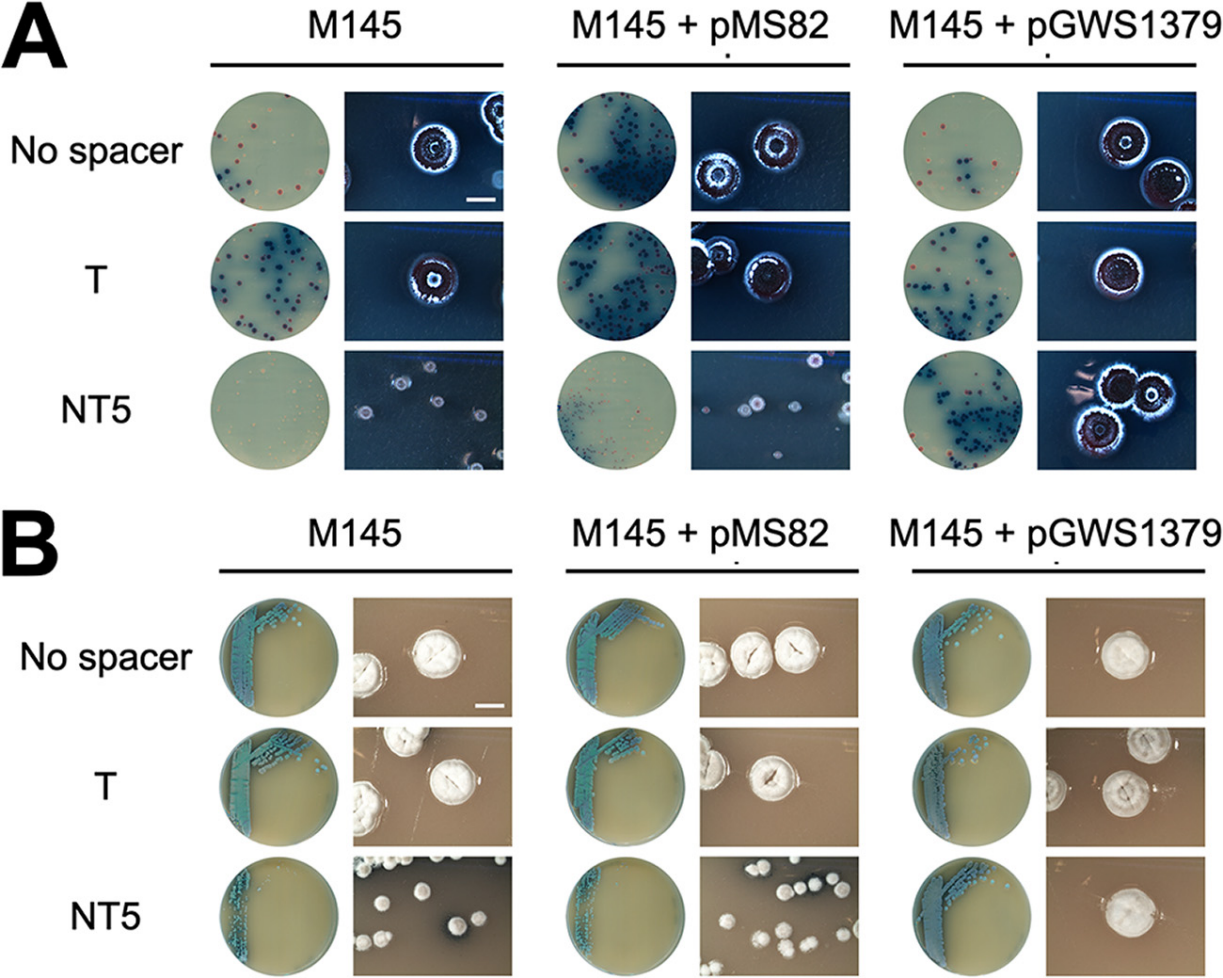
Ectopic expression of *mglA* allows silencing of *murG_Sco_* via CRISPRi. (A) CRISPRi constructs were introduced into S. coelicolor M145 or with the control plasmid pMS82 and into a recombinant strain with pGWS1379 integrated in its genome, thus expressing *K. viridifaciens* MglA. Expectedly, no effect was seen when we introduced CRISPRi constructs that either had no spacer or contained a spacer targeting the template strand (T) of *murG_Sco_.* However, constructs targeting the nontemplate strand (NT) resulted in severe phenotypic defects and sick colonies of S. coelicolor that lacked *mglA*, but not in pGWS1379 transformants that expressed *mglA*. (B) Morphology of colonies of the strains carrying the CRISPRi constructs after their transfer to fresh mannitol soya flour (MS) agar plates. Images were taken after 5 days of incubation at 30°C. Bar, 2 mm.

The observations that *mglA* can functionally replace *murG* in *K. viridifaciens* and that strains expressing only MglA produce wild-type peptidoglycan strongly suggest that the *mglA* gene product synthesizes lipid II. To further substantiate this, we investigated whether *mglA* could also functionally complement *murG* in another actinobacterium, namely, the model organism S. coelicolor M145, which itself does not harbor an orthologue of *mglA*. For this, we created the construct pGWS1379, expressing *mglA* from the constitutive modified ermE* promoter (78) in the integrative vector pMS82, and introduced it into S. coelicolor. As a control, we used the empty vector pMS82. We then applied CRISPR interference (CRISPRi) (45) to knock down the native *murG_Sco_* gene to assess viability. CRISPRi works only when the spacer of the endonuclease-deficient Cas9 (dCas9)/single guide RNA (sgRNA) complex targets the nontemplate strand of *murG_Sco_*, and not the template strand, or when the spacer is absent (45, 46). The functionality of the CRISPRi constructs was evident in control cells without *mglA*; colonies expressing the dCas9/sgRNA complex targeting the nontemplate strand of *murG_Sco_* in M145 formed small colonies, likely due to leaky expression of the essential *murG_Sco_* gene. Conversely, control transformants harboring CRISPRi constructs targeting the template strand or without the spacer (empty plasmid) grew normally (Fig. 6A). Excitingly, S. coelicolor transformants expressing *mglA* formed normal-size colonies under all conditions, even when *murG_Sco_* expression was knocked down by the CRISPRi system. Restoration of normal growth was also observed when these transformant colonies were transferred to fresh agar plates, while colonies of transformants lacking *mglA* remained small (Fig. 6B). This validates the concept that *mglA* of *K. viridifaciens* can functionally replace canonical *murG* in S. coelicolor. Taken together, our experiments show that the MglA enzyme can functionally replace the lipid II biosynthetic enzyme MurG, both in *Kitasatospora* and in *Streptomyces*.

## DISCUSSION

The cell wall is a hallmark feature of bacterial cells, and the steps involved in its biosynthesis are widely conserved across the bacterial domain. In all bacteria, the final cytosolic step in precursor biosynthesis is the conversion of lipid I to lipid II by MurG, encoded in the *dcw* gene cluster. We here show for the first time that the enzyme MglA can replace the activity of MurG and demonstrate that *murG* is dispensable in the filamentous actinomycete *K. viridifaciens* in the presence of *mglA*. MglA alone is sufficient to produce wild-type peptidoglycan. MglA is in fact widespread among the *Streptomycetaceae* and was identified in the genomes of 38% of all *Streptomyces* and *Kitasatospora* strains. Furthermore, introduction of *K. viridifaciens mglA* into S. coelicolor M145, which itself lacks an orthologue of *mglA*, allowed the knockdown of the canonical *murG* gene using CRISPRi, showing that the gene is a bona fide cell wall biosynthetic gene that is functional in different actinobacteria.

Filamentous actinomycetes are multicellular bacteria that form networks of interconnected hyphae, whereby sporulating aerial hyphae are established after a period of vegetative growth. *Streptomyces* is a wonderful model system for the study of cell division because, among other reasons, canonical cell division is not required for the normal growth of this bacterium (21, 25, 47). Most of the cell division proteins are encoded by genes located in the conserved *dcw* gene cluster. In streptomycetes, many cell division genes, such as *ftsI*, *ftsL*, *ftsW*, and *divIC*, are required only for sporulation and do not affect normal growth (48–50). Our data, surprisingly, show that many genes within the *dcw* cluster can be deleted simultaneously in *K. viridifaciens*, including *divIVA*, which is essential for polar growth in actinobacteria, by using a strain (*alpha*) with the ability to readily switch between a wall-deficient and filamentous mode of growth. The *alpha* strain thus provides a unique system for the identification of proteins that are required for polar growth. As a proof of concept for this principle, *divIVA*, which is required for polar growth, was successfully deleted. The absence of *divIVA* arrested growth in the wall-deficient state but had no effect on the synthesis of the PG building blocks, consistent with its role in driving apical growth. This indicates that the block in PG formation occurred in a later step of the PG biosynthesis pathway. Introduction of only *divIVA* in the *dcw* mutant restored polar growth, which was a rather surprising discovery given the absence of a whole string of genes involved in cell division and cell wall synthesis and, in particular, *murG*. MurG catalyzes the coupling of GlcNAc to lipid I, yielding the PG precursor lipid II, and this enzymatic activity is therefore essential for cell wall synthesis. The ability of *alpha* to produce a cell wall with an apparently normal architecture, as shown by the analysis of the peptidoglycan, indicated that *K. viridifaciens* possesses other enzymes capable of synthesizing lipid II in the absence of *murG*. An *in silico* search in the genome of *K. viridifaciens* identified *mglA* (BOQ63_RS12640), which is a distant relative of MurG with the likely ability to replace the activity of canonical MurG. This is based on, among other things, the presence of the two domains that are known to be required for the transfer of GlcNAc to lipid I. Many actinobacteria possess proteins carrying these two domains, suggesting that MglA proteins are common in these bacteria. In fact, some species even contain three genes for MurG-like proteins, in addition to the canonical MurG encoded in the *dcw* gene cluster. Interestingly, both *murG* and *mglA* could be individually deleted in the wild-type strain, whereby the resulting mutants showed normal growth and development when strains were grown in nonstressed environments. However, the *murG* mutant was more susceptible to cell wall-targeting antibiotics than the wild-type strain or its *mglA* mutant. Considering that MglA alone suffices to produce normal peptidoglycan, this suggests that MurG is required to build a more robust cell wall. Deletion of *murG* was possible only after exposing transformants to hyperosmotic growth conditions. We hypothesize that the hyperosmotic conditions activated the transcription of *mglA*, thus allowing deletion of *murG* specifically under these growth conditions. This implies that the function of *mglA* is to synthesize lipid II under specific growth conditions, for instance during hyperosmotic stress.

In further support of the function of MglA as an alternative lipid II synthase, we tested if it could also take over the function of *murG* in another bacterium. For this, we chose the model streptomycete S. coelicolor M145, which is a distinct genus within the *Streptomycetaceae* (31, 51) but lacks a copy of *mglA*. Importantly, *murG* could be readily depleted using CRISPRi in strains expressing *mglA* from a constitutive promoter, while knockdown of *murG* in colonies of S. coelicolor harboring control plasmids led to very severe growth defects. This not only validates our data that *mglA* encodes a lipid II synthase but also indicates that this is a more universal phenomenon that does not occur only in specific strains of *Kitasatospora* or connect to strains that have the capacity to produce natural wall-less cells. Furthermore, it shows that no additional *Kitasatospora* genes are required to allow *mglA* to functionally complement *murG* in *Streptomyces*.

We also attempted to delete *murG* and *mglA* simultaneously in *alpha*. While the single mutants were readily obtained, we never obtained strains that were completely devoid of both *murG* and *mglA*, despite many attempts. Like mycelia, L-forms are multinucleated cells, and some cells of the population retained *mglA*, most likely to ensure minimal levels of lipid II. Consistent with this idea is the finding that antibiotics that target lipid II, such as vancomycin, are lethal to *alpha* (our unpublished data). We hypothesize that this lethality is caused by depletion of the lipid carrier undecaprenyl diphosphate, which is also used in other pathways and which may be essential for these L-forms. Removing *mglA* in strains lacking *murG* virtually blocked the ability to switch to the filamentous mode of growth, whereas each of the single mutants switched as efficiently as the parental *alpha* strain. Thus, we show that MglA is an enzyme involved in cell wall metabolism, which appears to facilitate switching between a wall-deficient and a walled lifestyle.

## MATERIALS AND METHODS

### Strains and media.

Bacterial strains used in this study are shown in Table 3. To obtain sporulating cultures of *K. viridifaciens* and S. coelicolor, strains were grown at 30°C for 4 days on MYM (52). For general cloning purposes, E. coli strains DH5α and JM109 were used, while E. coli ET12567 and SCS110 were used to obtain unmethylated DNA. E. coli strains were grown at 37°C in LB medium supplemented with chloramphenicol (25 μg ml^−1^), ampicillin (100 μg ml^−1^), apramycin (50 μg ml^−1^), kanamycin (50 μg ml^−1^), or viomycin (30 μg ml^−1^), where necessary.

**TABLE 3:** Strains used in this study

Strains	Genotype	Reference or source
E. coli strains
DH5α	F^–^ ϕ?80*lacZ*ΔM15 Δ(*lacZYA-argF*)*U169 recA1 endA1 hsdR17*(r_K_^–^ m_K_^–^) *phoA supE44 thi-1 gyrA96 relA1* λ^–^	73
JM109	*recA1 endA1 gyrA96 thi hsdR17 supE44 relA1* λ- Δ(*lac-proAB*) [F′ *traD36 proAB* Δ(*lacI*^q^*Z*ΔM15]	74
ET12567	F^–^ *dam-13*::Tn*9 dcm-6 hsdM hsdR recF143 zjj-202*::Tn*10 galK2 galT22 ara14 lacY1 xyl-5 leuB6 thi-1 tonA31 rpsL136 hisG4 tsx-78 mtl-1 glnV44*	75
SCS110	*rpsL* (Str^r^) *thr leu endA thi-1 lacY galK galT ara tonA tsx dam dcm supE44* Δ(*lac-proAB*) [*F’ traD36 proAB lacI*^q^ *lacZ*ΔM15]	76

Actinobacteria
S. coelicolor A3(2) M145	Wild-type strain	Lab collection
M145(pGWS1379)	S. coelicolor A3(2) M145 expressing *mglA*	This work
*K. viridifaciens* DSM40239	Wild-type strain	DSMZ
DSM40239 Δ*murG*	*K. viridifaciens* DSM40239 in which *murG* is replaced by the *aac(3)IV* apramycin resistance cassette	This work
DSM40239 Δ*mglA*	*K. viridifaciens* DSM40239 in which *mglA* is replaced by the *aac(3)IV* apramycin resistance cassette	This work

*K. viridifaciens* L-form strains
*alpha*	L-form cell line obtained after induction with penicillin and lysozyme	32
*alpha*(pKR1)	*alpha* carrying pKR1	This work
*alpha*(pKR2)	*alpha* carrying pKR2	This work
*alpha*(pGreen)	*alpha* constitutively expressing eGFP	This work
Δ*divIVA* strain	*alpha* in which *divIVA* is replaced by the *aac(3)IV* apramycin resistance cassette	This work
Δ*dcw* strain	*alpha* in which *ftsW*, *murG*, *ftsQ*, *ftsZ*, *ylmD*, *ylmE*, *sepG*, *sepF*, and *divIVA* are replaced by the *aac(3)IV* apramycin resistance cassette	This work
Δ*divIVA*/*divIVA* strain	*divIVA* mutant containing *divIVA* expressed from the *gap1* promoter	This work
Δ*dcw divIVA* strain	*dcw* mutant containing *divIVA* expressed from the *gap1* promoter	This work
Δ*murG* strain	*alpha* in which *murG* is replaced by the *aac(3)IV* apramycin resistance cassette	This work
Δ*mglA* strain	*alpha* in which *mglA* is replaced by the *aac(3)IV* apramycin resistance cassette	This work
Δ*murG* Δ*mglA* strain (merodiploid)	Δ*murG* strain in which *mglA* is replaced by the *vph* viomycin resistance cassette	This work

To support the growth of wall-deficient cells, strains were grown in liquid LPB medium while being shaken at 100 rpm or grown on solid LPMA medium at 30°C (32). To switch from the wall-deficient to the filamentous mode of growth, L-form colonies grown on LPMA for 7 days were streaked on MYM. If needed, mycelial colonies of switched strains were transferred after 4 days to liquid tryptic soy broth sucrose (TSBS) medium and grown for 2 days at 30°C, while being shaken at 200 rpm.

### Construction of plasmids.

All plasmids and primers used in this work are shown in Tables 4 and 5, respectively.

**TABLE 4:** Vectors and constructs used in this study

Plasmid	Description and relevant features	Reference
pWHM3	Unstable, multicopy and self-replicating S*treptomyces* vector; contains thiostrepton and ampicillin resistance cassette.	56
pIJ780	Plasmid containing a viomycin (*vph*) resistance cassette	54
pIJ8600	E. coli-*Streptomyces* shuttle vector containing the ϕC31 *attP-int* region for genomic integration; confers resistance to apramycin and thiostrepton	53
pIJ8630	E. coli-*Streptomyces* shuttle vector containing the ϕC31 *attP-int* region for genomic integration; confers resistance to apramycin	53
pSET152	E. coli-*Streptomyces* shuttle vector; high copy number in E. coli and integrative in *Streptomyces*	77
pHM10a	Conjugative E. coli*-Streptomyces* shuttle vector, harboring P*ermE* and a ribosome binding site	58
pMS82	E. coli-*Streptomyces* shuttle vector; high copy number in E. coli and integrative in *Streptomyces*	59
pGreen	pIJ8630 containing the eGFP gene under the control of the constitutive *gap1* promoter of S. coelicolor	43
pKR1	pIJ8630 derivative containing the viomycin resistance cassette from pIJ780 cloned into the unique NheI site	This work
pKR2	pKR1 derivative containing a C-terminal eGFP gene fusion to *divIVA* of *K. viridifaciens* under the control of the *gap1* promoter of S. coelicolor	This work
pKR3	pWHM3 containing the flanking regions of the *K. viridifaciens divIVA* gene interspersed with the *apra-loxP* cassette	This work
pKR4	pWHM3 derivative containing the flanking regions around the *K. viridifaciens* partial *dcw* gene cluster (*ftsW*, *murG*, *ftsQ*, *ftsZ*, *ylmD*, *ylmE*, *sepF*, *sepG*, *divIVA*) interspersed with the *apra-loxP* cassette	This work
pKR5	pIJ8600 derivative containing the *gap1* promoter of S. coelicolor	This work
pKR6	pKR5 derivative containing the *divIVA* gene of *K. viridifaciens* under the control of the *gap1* promoter of S. coelicolor	This work
pKR8	pWHM3 containing the flanking regions of the *K. viridifaciens murG* gene interspersed with the *apra-loxP* cassette	This work
pKR9	pWHM3 containing the flanking regions of the *K. viridifaciens mglA* (BOQ63_RS12640) gene interspersed with the *apra-loxP* cassette	This work
pKR10	pWHM3 containing the flanking regions of the *K. viridifaciens mglA* (BOQ63_RS12640) gene interspersed with the viomycin resistance cassette	This work
pGWS1369	pSET152 lacking its NcoI site	This work
pGWS1370	pGWS1369 containing an sgRNA scaffold (no spacer) and P*gapdh-dCas9*	This work
pGWS1371	pWGS1370 containing a spacer targeting the template strand of *SCO2084*	This work
pGWS1376	pWGS1370 containing a spacer targeting the nontemplate strand of *SCO2084*	This work
pGWS1378	pSET152 containing P*ermE-mglA*	This work
pGWS1379	pMS82 containing P*ermE-mglA*	This work

**TABLE 5:** Primers used in this study

Primer	Sequence (5′–3′)
*vph*-FW-NheI	GACGCTAGCGGCTGACGCCGTTGGATACACCAAG
*vph*-RV-NheI	GACGCTAGCAATCGACTGGCGAGCGGCATCCTAC
P_Gap1_-FW-BglII	GATTACAGATCTCCGAGGGCTTCGAGACC
P_Gap1_-RV-XbaI	GATGACTCTAGACCGATCTCCTCGTTGGTAC
*divIVA*-FW-XbaI	GTCAAGCTTCTAGAATGCCATTGACCCCCGAGGA
*divIVA*-Nostop-RV-NdeI	GACCATATGGTTGTCGCCGTCCTCGTCAATCAGG
P1-*divIVA*-FW	GACGACGAATTCTGTGATGACCGTCGCTCCACTG
P2-*divIVA*-RV	GACGACTCTAGACTTCCGCATGTTGGCCTGGTTC
P1-*dcw*-FW	GACGAATTCTCCGCGAGGTCACGTACATC
P2-*dcw*-RV	GACTCTAGAAGAGCACCAGTGCGAGCTTG
P3-*dcw*-FW	GACTCTAGAAGCAGCAGATGGGCAACCAG
P4-*dcw*-RV	GATAAGCTTCCCGGCTACAACCTCAGTTGTC
Delcheck-*divIVA*-FW	TGACCCGGCCACGACTTTAC
Delcheck-*divIVA*-RV	GGACGCCCTCAACAAAC
Delcheck-*dcw*-FW	CCAGAACTGGCTGGATTTCG
Delcheck-*dcw*-RV	GTCTCCAGGTACGACTTCAG
*divIVA*-XbaI-FW	GTCAAGCTTCTAGAATGCCATTGACCCCCGAGGA
*divIVA*-NdeI-RV	GATCGAATTCATATGCCCGGCTACAACCTCAGTTGTC
*divIVA* seq1-FW	AGCAGCAGATGGGCAACCAG
*divIVA* seq2-FW	CGCGTCTGAAGTCGTACCTG
*divIVA* seq-RV	ACCTCGTCCTCGTCATAGC
SCO2079_F-520	TCACGGCGCTGTCGAAGGAGGCCG
SCO2079_R + 1162	CTCATCGAGGAAGGCATCGACCTC
*divIVA_Sco_*-FW	AAGGCTACGCCGTACTACAG
*divIVA_Sco_*-RV	AGATACGGGCTTGCCGAATG
P1-*murG*-Fw	CATCGAATTCGATATCTTTCGGCTTCTTCCAGTTCC
P2-*murG*-Rv	CATCCATGTCTAGACGACATGCACCGAAATTCAC
P3-*murG*-Fw	CATCCATGTCTAGATGGTGTACGAGGCGATCCAG
P4-*murG*-Rv	CATGGATATCAAGCTTGACGGATGTCGATGGGTAGG
Delcheck-*murG*-Fw	AGCAAGAACTCCCGGATCAG
Delcheck-*murG*-Rv	AGCACCGACGAGAAGAACAC
P1-*mglB*-Fw	CTGAGAATTCGATATCTTCTCGTGGGAACACCGGGCA
P2-*mglB*-Rv	CTGATCTAGAGGTGACGATCAGCCGCATAGG
P3-*mglB*-Fw	CTGATCTAGAGACCGTCTCGTGGACGTGCTG
P4-*mglB*-Rv	CTGAAAGCTTGATATCGTTCCCGCTACCCGAACGGAAC
Delcheck-*mglA*-Fw	CTGAATGTTCCAAGCGTGAACCGGGA
Delcheck-*mglA-*Rv	CTGAGCGACTACAAGGCGTACCAGG
*vph*-Fw-EcoRI-HindIII-XbaI	GACGAATTCAAGCTTTCTAGAGGCTGACGCCGTTGGATACACCAAG
*vph*-Rv-EcoRI-HindIII-XbaI	GACGAATTCAAGCTTTCTAGAAATCGACTGGCGAGCGGCATCCTAC
152DNcoI_F	GCAAGCCATTCTGTCCGCGATGGACAAGCTGTACT
152DNcoI_R	GCAGTACAGCTTGTCCATCGCGGACAGAATGGCTT
SgTermi_R_B	CTAGGGATCCCAAAAAACCCCTCAAGACCCGTTTAGAGGCCCCAAGGGGTTATGCTAGTTACGCCTACGTAAAAAAAGCACCGACTCGGTGCC
SCO2084_T_F	CATGCCATGGACCGTGGGGATCACGGCCCTGTTTTAGAGCTAGAAATAGC
SCO2084_NT5_F	CATGCCATGGTTGGCCTCGTGCACGACGATGTTTTAGAGCTAGAAATAGC
mglA_F+4_ENdeI	CTGAGAATTCCATATGCGGCTGATCGTCACCGGCG
mglA_R+1146_HX	CTGAAAGCTTTCTAGACTAGCGGTCCACTACCGACAGCAGCAC

### (i) Construction of the DivIVA localization construct pKR2.

To localize DivIVA, we first created plasmid pKR1 containing a viomycin resistance cassette cloned into the unique NheI site of pIJ8630 (53). To this end, the viomycin resistance cassette was amplified from pIJ780 (54) with the primers *vph*-FW-NheI and *vph*-RV-NheI. Next, we amplified the constitutive *gap1* promoter as a 450-bp fragment from the genome of S. coelicolor with the primers P*gap1*-FW-BglII and P*gap1*-RV-XbaI. We also amplified the *divIVA* coding sequence (the bp +1 to +1335 region relative to the start codon of *divIVA* [BOQ63_RS32500]) from the chromosome of *K. viridifaciens* using primers *divIVA*-FW-XbaI and *divIVA*-Nostop-RV-NdeI (55). Finally, the promoter and *divIVA* coding sequences were cloned into pKR1 as BglII/XbaI and XbaI/NdeI fragments, respectively, yielding plasmid pKR2.

### (ii) Construction of the deletion constructs pKR3, pKR4, pKR8, pKR9, and pKR10.

The *divIVA* mutant was created in *K. viridifaciens* using pKR3, which is a derivative of the unstable plasmid pWHM3 (56). In the *divIVA* mutant, nucleotides +205 to +349 relative to the start codon of *divIVA* were replaced with the *loxP-apra* resistance cassette as described previously (57). A similar strategy was used for the deletion of the partial *dcw* cluster (plasmid pKR4) and for the deletion of *murG* (plasmid pKR8) and *mglA* (plasmid pKR9). For the deletion of the partial *dcw* cluster, the chromosomal region from bp +487 relative to the start of the *ftsW* gene (BOQ63_RS32460) until bp +349 relative to the start of the *divIVA* gene was replaced with the apramycin resistance marker. For the deletion of *murG* (BOQ63_RS32465, located in the *dcw* cluster), bp +10 to +1077 relative to the start codon of *murG* were replaced with the *loxP-apra* resistance cassette, while for the *mglA* (BOQ63_RS12640) deletion, the chromosomal region from bp +18 to +1105 relative to the start of *mglA* was replaced with the apramycin resistance marker. To construct the *murG mglA* double mutant, pKR10 was created, replacing the apramycin resistance cassette in pKR8 by a viomycin resistance cassette. To this end, the viomycin resistance cassette was amplified from pIJ780 (54) with the primers *vph*-Fw-EcoRI-HindIII-XbaI and *vph*-Rv-EcoRI-HindIII-XbaI. The viomycin resistance cassette contained on the PCR fragment was then cloned into pKR8 using XbaI, thereby replacing the apramycin cassette and yielding pKR10.

### (iii) Construction of the complementation constructs pKR6 and pKR7.

For complementation of *divIVA* under the control of the strong *gap1* promoter (43), construct pKR6 was made. First, we created plasmid pKR5 with the strong *gap1* promoter. The promoter region of *gap1* (SCO1947) was amplified with the primers P*gap1*-FW-BglII and P*gap1*-RV-XbaI using S. coelicolor genomic DNA as the template. Next, the *gap1* promoter was cloned as a BglII/XbaI fragment into the integrative vector pIJ8600 (53) to generate plasmid pKR5. Afterwards, the *divIVA* coding sequence was amplified from the genome of *K. viridifaciens* with the primers *divIVA*-XbaI-FW and *divIVA*-NdeI-RV. Finally, to create the plasmid pKR6, the XbaI/NdeI fragment containing the *divIVA* coding sequence was cloned in pKR5.

### (iv) Construction of the *mglA* expression construct pGWS1379.

A DNA fragment containing the modified ermE* promoter was obtained as an EcoRI/NdeI fragment from pHM10a (58), while *mglA* was amplified by PCR from *K. viridifaciens* chromosomal DNA using the primer pair mglA_F+4_ENdeI and mglA_R+1146_HX. The ermEp* promoter fragment and NdeI/XbaI-digested *mglA* were simultaneously cloned into EcoRI/XbaI-digested pSET152 to generate construct pGWS1378. The insert of pGWS1378 was then introduced as a PvuII fragment into EcoRV-digested pMS82 (59) to generate construct pGWS1379. This construct was then introduced into S. coelicolor M145 via protoplast transformation as described previously (60).

### Transformation of L-forms.

Transformation of *alpha* essentially followed the protocol for the rapid small-scale transformation of *Streptomyces* protoplasts (60), with the difference that cells (50 μl) from a mid-exponential growing L-form culture were used instead of protoplasts. Typically, 1 μg DNA was used for each transformation. Transformants were selected by applying an overlay containing the required antibiotics in P buffer after 20 h. Further selection of transformants was done on LPMA medium supplemented with apramycin (50 μg ml^−1^), thiostrepton (5 μg ml^−1^), or viomycin (30 μg ml^−1^), when necessary. Transformants were verified by PCR.

### *murG_Sco_* (SCO2084) knockdown via CRISPRi.

The NcoI restriction site within the integrase gene of phage ϕC31 in pSET152 was removed by introducing a silent GCC-to-GCG change in codon A360 via site-directed mutagenesis by PCR using primer pairs 152DNcoI_F and 152DNcoI_R to generate construct pGWS1369. Subsequently, a DNA fragment containing the sgRNA scaffold (no spacer) and P*gapdh-dcas9* of construct pGWS1049 (46) was cloned as an EcoRI/XbaI fragment into pGWS1369 to generate construct pGWS1370. The 20-nt spacer sequence was introduced into the sgRNA scaffold by PCR using forward primer SCO2084_T_F or SCO2084_NT5_F together with the reverse primer SgTermi_R_B. The PCR products were cloned as NcoI/BamHI fragments into pGWS1370 to generate constructs pGWS1371 (targeting the template strand of SCO2084) and pGWS1376 (targeting the nontemplate strand of SCO2084). Constructs pGWS1370 (no spacer), pGWS1371 (targeting the template strand), and pGWS1376 (targeting the nontemplate strand) were introduced into S. coelicolor M145(pMS82) (empty plasmid) and M145(pGWS1379) (expressing *mglA*) via protoplast transformation as described previously (60).

### Microscopy.

Strains grown in LPB or LPMA were imaged using a Zeiss Axio Lab A1 upright microscope equipped with an AxioCam MRc digital camera. A thin layer of LPMA (without horse serum) was applied to the glass slides to immobilize the cells prior to the microscopic analysis.

### (i) Fluorescence microscopy.

Fluorescence microscopy pictures were obtained with a Zeiss Axioscope A1 upright fluorescence microscope equipped with an AxioCam MRc5 camera. Aliquots of 10 μl of live cells were immobilized on top of a thin layer of LPMA (without horse serum) prior to analysis. Fluorescent images were obtained using a 470/40-nm-band-pass excitation and a 505/560-nm-band-pass detection, using an 100×-numerical-aperture 1.3 objective. To obtain a sufficiently dark background, the background of the images was set to black. These corrections were made using Adobe Photoshop CS5.

### (ii) Time-lapse microscopy.

To visualize the proliferation of *alpha*, cells were collected and resuspended in 300 μl LPB (containing 4 to 22% sucrose) and placed in the wells of a chambered 8-well μ-slide (ibidi). Cells were imaged on a Nikon Eclipse Ti-E inverted microscope equipped with a confocal spinning disk unit (CSU-X1) operated at 10,000 rpm (Yokogawa), using a 40× Plan Fluor lens (Nikon), and illuminated in bright field. Images were captured every 2 min for 10 to 15 h by an Andor iXon Ultra 897 high-speed electron microscope charge-coupled device (EM-CCD) camera (Andor Technology). Z-stacks were acquired at 0.2- to 0.5-μm intervals using an NI-DAQ-controlled Piezo element. During imaging, wall-less cells were kept at 30°C using an INUG2E-TIZ stage top incubator (Tokai Hit).

### (iii) Electron microscopy.

For transmission electron microscopy, L-forms obtained from a 7-day-old liquid-grown *alpha* culture were trapped in agarose blocks prior to fixation with 1.5% glutaraldehyde and a postfixation step with 1% OsO_4_. Samples were embedded in Epon and sectioned into 70-nm slices. Samples were stained using uranyl acetate (2%) and lead citrate (0.4%), if necessary, before being imaged using a JEOL 1010 or an FEI Tecnai 12 BioTWIN transmission electron microscope.

### DivIVA detection using Western analysis.

To detect DivIVA using Western analysis, the biomass of L-form strains was harvested after 7 days of growth in LPB medium, while the biomass of mycelial strains was obtained from liquid-grown TSBS cultures after 17 h. Cell pellets were washed twice with 10% phosphate-buffered saline (PBS), after which they were resuspended in 50 mM HEPES, pH 7.4, 50 mM NaCl, 0.5% Triton X-100, 1 mM PFMS (phenylmethylsulfonyl fluoride), and P8465 protease inhibitor cocktail (Sigma). The cells and mycelia were disrupted with a Bioruptor Plus sonication device (Diagenode). Complete lysis was verified by microscopy, after which the soluble cell lysate was separated from the insoluble debris by centrifugation at 13,000 rpm for 10 min at 4°C. The total protein concentration in the cell lysates was quantified by a bicinchoninic acid (BCA) assay (Sigma-Aldrich). Equal amounts of total proteins were separated with SDS-PAGE using 12.5% gels. Proteins were transferred to polyvinylidene difluoride (PVDF) membranes (GE Healthcare) with the Mini Trans-Blot Cell (Bio-Rad Laboratories) according to the manufacturer’s instructions. DivIVA was detected using a 1:5,000 dilution of polyclonal antibodies raised against Corynebacterium glutamicum DivIVA (kindly provided by Marc Bramkamp). The secondary antibody, anti-rabbit IgG conjugated to alkaline phosphatase (Sigma), was visualized with the BCIP (5-bromo-4-chloro-3-indolylphosphate)-NBT (nitroblue tetrazolium) color development substrate (Promega).

### Isolation of cytoplasmic peptidoglycan precursors.

For the cytoplasmic PG precursor isolation and identification, we used a modification of the method previously described (61). The *alpha* strain and the *divIVA* and *dcw* mutants were grown in LPB for 7 days, while the wild-type *K. viridifaciens* strain was grown for 3 days in a modified version of LPB lacking sucrose. The cells were harvested by centrifugation at 4°C and washed in 0.9% NaCl. Cells were extracted with 5% cold trichloric acid (TCA) for 30 min at 4°C. The extracts were centrifuged at 13,000 rpm for 5 min at 4°C, after which the supernatants were desalted on a Sephadex G-25 column (Illustra NAP-10 columns; GE Healthcare, Pittsburgh, PA) and concentrated by rotary evaporation. The concentrated precursors were dissolved in 200 μl high-performance liquid chromatography (HPLC)-grade water.

### Peptidoglycan extraction.

The peptidoglycan architecture was analyzed as described previously (62). Mycelia of the wild-type strain, *alpha*, and the *dcw* mutant complemented with *divIVA* were grown on top of cellophane discs on modified LPMA medium lacking sucrose and horse serum. Following growth, the mycelial mass was removed from the cellophane, washed in 0.1 M Tris-HCl, pH 7.5, and lyophilized. Ten milligrams of the lyophilized biomass was used for PG isolation. Therefore, the biomass was boiled in 0.25% SDS in 0.1 M Tris-HCl, pH 6.8, thoroughly washed, sonicated, and treated with DNase, RNase, and trypsin. Inactivation of these enzymes was performed by boiling the samples, followed by washing them with water. Wall teichoic acids were removed with 1 M HCl (63). PG was digested with mutanolysin and lysozyme. Muropeptides were reduced with sodium borohydride, and the pH was adjusted to 3.5 to 4.5 with phosphoric acid.

### LC-MS analysis of PG precursors and muropeptides.

The LC-MS setup consisted of a Waters Acquity UPLC system (Waters, Milford, MA, USA) and an LTQ Orbitrap XL hybrid ion Trap-Orbitrap mass spectrometer (Thermo Fisher Scientific, Waltham, MA, USA) equipped with an Ion Max electrospray source. Chromatographic separation of muropeptides and precursors was performed on an Acquity ultraperformance LC (UPLC) HSS T3 C_18_ column (1.8 μm, 100 Å, 2.1 by 100 mm). Mobile phase A consisted of 99.9% H_2_O and 0.1% formic acid, while mobile phase B consisted of 95% acetonitrile, 4.9% H_2_O, and 0.1% formic acid. All solvents used were of LC-MS grade or better. The flow rate was set to 0.5 ml min^−1^. The binary gradient program consisted of 1 min of 98% phase A, 12 min of from 98% A to 85% A, and 2 min of from 85% A to 0% A. The column was then flushed for 3 min with 100% phase B, after which the gradient was set to 98% and the column was equilibrated for 8 min. The column temperature was set to 30°C, and the injection volume used was 5 μl. The temperature of the autosampler tray was set to 8°C. Data were collected in the positive electrospray ionization (ESI) mode, with a scan range of *m/z* 500 to 2,500 in high-range mode. The resolution was set to 15,000 (at *m/z* 400).

### Sequence homology analysis of *dcw* gene clusters.

The homology search of the different *dcw* clusters was done using MultiGeneBlast (64). The query used for the search was the *dcw* cluster from Streptomyces coelicolor A3(2), for which the required sequences were obtained from the *Streptomyces* Annotation Sever (StrepDB). The homology search included the loci from SCO2077 (*divIVA*) to SCO2091 (*ftsL*). A database was constructed with genome assemblies obtained from the NCBI. The analyzed species have the following NCBI accession numbers: AL645882.2 [S. coelicolor A3(2)], NZ_MPLE00000000.1 (*Kitasatospora viridifaciens* DSM40239), CP000480 (Mycobacterium smegmatis MC2 155), AL123456 (Mycobacterium tuberculosis H37Rv), CP014279 (Corynebacterium stationis ATCC 6872), BX927147 (Corynebacterium glutamicum ATCC 13032), AL009126 (Bacillus subtilis subsp.168), U00096 (Escherichia coli K-12), CP000253.1 (Staphylococcus aureus NTC8325), and AE007317 (Streptococcus pneumoniae R6). In the homology search, the BLAST parameters were set to a minimal sequence coverage of 25% and a minimal identity of 30%. The first 11 hits of the MultiGeneBlast output are shown in Fig. S1, where homologue genes are represented by arrows with the same colors.

### Phylogeny analysis of *Streptomyces* and *Kitasatospora* species.

A set of 1,050 *Streptomyces* and *Kitasatospora* genomes was downloaded from the NCBI database by querying the fasta files in combination with the taxonomic identifier. To this set, 116 unpublished draft genome sequences of an in-house collection of actinomycetes were added (65). Complete protein sets encoded within the genomes of *Streptomyces* and *Kitasatospora* spp. were extracted. The Pfam domains of four housekeeping proteins, AtpD (ATP synthase subunit beta), RecA (recombinase A), TrpB (tryptophan synthase beta chain), and GyrB (DNA gyrase subunit B), were retrieved from https://pfam.xfam.org/ and are annotated as PF00213, PF00154, PF06233, and PF00204, respectively. Using the selected Pfam domains, the Hmmsearch program of the HMMER v3.0 package (66) was employed to identify analogous proteins within the chosen species. MAFFT was used to perform a multiple-sequence alignment (67). Aligned sequences were concatenated using SeqKit (68), and maximum-likelihood phylogenetic trees were calculated with RAxML (69). iTOL (70) was used for the visualization of the phylogenetic tree.

### Detection of *murG* genes in *Streptomyces* and *Kitasatospora* species.

MurG domains were predicted using the Pfam database (44). Proteins with the predicted MurG domains were used to search in the complete protein sets encoded within the extracted genomes using Hmmsearch. Instead of a multiple-sequence alignment, each protein domain sequence was aligned to its profile hidden Markov model from Pfam using the hmmalign tool (71). For each protein, a pairwise distance was calculated for all detected MurG proteins, and the threshold was set at 0.9. Network visualizations were constructed using Cytoscape (v. 3.7.1) (72).

## References

[1] Liu Y, Breukink E. 2016. The membrane steps of bacterial cell wall synthesis as antibiotic targets. Antibiotics (Basel) 5:28. doi:10.3390/antibiotics5030028. PMC5039524 27571111

[2] Mohammadi T, van Dam V, Sijbrandi R, Vernet T, Zapun A, Bouhss A, Diepeveen-de Bruin M, Nguyen-Distèche M, de Kruijff B, Breukink E. 2011. Identification of FtsW as a transporter of lipid-linked cell wall precursors across the membrane. EMBO J 30:1425–1432. doi:10.1038/emboj.2011.61. 21386816 PMC3102273

[3] Sham LT, Butler EK, Lebar MD, Kahne D, Bernhardt TG, Ruiz N. 2014. MurJ is the flippase of lipid-linked precursors for peptidoglycan biogenesis. Science 345:220–222. doi:10.1126/science.1254522. 25013077 PMC4163187

[4] Meeske AJ, Sham LT, Kimsey H, Koo BM, Gross CA, Bernhardt TG, Rudner DZ. 2015. MurJ and a novel lipid II flippase are required for cell wall biogenesis in *Bacillus subtilis*. Proc Natl Acad Sci U S A 112:6437–6442. doi:10.1073/pnas.1504967112. 25918422 PMC4443310

[5] Scheffers DJ, Pinho MG. 2005. Bacterial cell wall synthesis: new insights from localization studies. Microbiol Mol Biol Rev 69:585–607. doi:10.1128/MMBR.69.4.585-607.2005. 16339737 PMC1306805

[6] Meeske AJ, Riley EP, Robins WP, Uehara T, Mekalanos JJ, Kahne D, Walker S, Kruse AC, Bernhardt TG, Rudner DZ. 2016. SEDS proteins are a widespread family of bacterial cell wall polymerases. Nature 537:634–638. doi:10.1038/nature19331. 27525505 PMC5161649

[7] Cho H, Wivagg CN, Kapoor M, Barry Z, Rohs PD, Suh H, Marto JA, Garner EC, Bernhardt TG. 2016. Bacterial cell wall biogenesis is mediated by SEDS and PBP polymerase families functioning semi-autonomously. Nat Microbiol 1:16172. doi:10.1038/nmicrobiol.2016.172. 27643381 PMC5030067

[8] Pazos M, Peters K, Vollmer W. 2017. Robust peptidoglycan growth by dynamic and variable multi-protein complexes. Curr Opin Microbiol 36:55–61. doi:10.1016/j.mib.2017.01.006. 28214390

[9] Vicente M, Errington J. 1996. Structure, function and controls in microbial division. Mol Microbiol 20:1–7. doi:10.1111/j.1365-2958.1996.tb02482.x. 8861198

[10] Tamames J, González-Moreno M, Mingorance J, Valencia A, Vicente M. 2001. Bringing gene order into bacterial shape. Trends Genet 17:124–126. doi:10.1016/s0168-9525(00)02212-5. 11226588

[11] Mingorance J, Tamames J, Vicente M. 2004. Genomic channeling in bacterial cell division. J Mol Recognit 17:481–487. doi:10.1002/jmr.718. 15362108

[12] Claessen D, Rozen DE, Kuipers OP, Søgaard-Andersen L, van Wezel GP. 2014. Bacterial solutions to multicellularity: a tale of biofilms, filaments and fruiting bodies. Nat Rev Microbiol 12:115–124. doi:10.1038/nrmicro3178. 24384602

[13] Flärdh K, Buttner MJ. 2009. *Streptomyces* morphogenetics: dissecting differentiation in a filamentous bacterium. Nat Rev Microbiol 7:36–49. doi:10.1038/nrmicro1968. 19079351

[14] Barka EA, Vatsa P, Sanchez L, Gaveau-Vaillant N, Jacquard C, Meier-Kolthoff JP, Klenk H-P, Clément C, Ouhdouch Y, van Wezel GP. 2016. Taxonomy, physiology, and natural products of *Actinobacteria*. Microbiol Mol Biol Rev 80:1–43. doi:10.1128/MMBR.00019-15. 26609051 PMC4711186

[15] Bérdy J. 2012. Thoughts and facts about antibiotics: where we are now and where we are heading. J Antibiot (Tokyo) 65:385–395. doi:10.1038/ja.2012.27. 22511224

[16] Celler K, Koning RI, Willemse J, Koster AJ, van Wezel GP. 2016. Cross-membranes orchestrate compartmentalization and morphogenesis in *Streptomyces*. Nat Commun 7:ncomms11836. doi:10.1038/ncomms11836. 27291257 PMC4909990

[17] Wildermuth H, Hopwood DA. 1970. Septation during sporulation in *Streptomyces coelicolor*. J Gen Microbiol 60:51–59. doi:10.1099/00221287-60-1-51. 5488466

[18] Manteca A, Fernandez M, Sanchez J. 2005. A death round affecting a young compartmentalized mycelium precedes aerial mycelium dismantling in confluent surface cultures of *Streptomyces antibioticus*. Microbiology (Reading) 151:3689–3697. doi:10.1099/mic.0.28045-0. 16272390

[19] Tenconi E, Traxler MF, Hoebreck C, van Wezel GP, Rigali S. 2018. Production of prodiginines is part of a programmed cell death process in *Streptomyces coelicolor*. Front Microbiol 9:1742. doi:10.3389/fmicb.2018.01742. 30127771 PMC6087738

[20] Schwedock J, McCormick JR, Angert ER, Nodwell JR, Losick R. 1997. Assembly of the cell division protein FtsZ into ladder-like structures in the aerial hyphae of *Streptomyces coelicolor*. Mol Microbiol 25:847–858. doi:10.1111/j.1365-2958.1997.mmi507.x. 9364911

[21] Jakimowicz D, van Wezel GP. 2012. Cell division and DNA segregation in *Streptomyces*: how to build a septum in the middle of nowhere? Mol Microbiol 85:393–404. doi:10.1111/j.1365-2958.2012.08107.x. 22646484

[22] McCormick JR, Losick R. 1996. Cell division gene *ftsQ* is required for efficient sporulation but not growth and viability in *Streptomyces coelicolor* A3(2). J Bacteriol 178:5295–5301. doi:10.1128/jb.178.17.5295-5301.1996. 8752351 PMC178330

[23] Edwards DH, Errington J. 1997. The *Bacillus subtilis* DivIVA protein targets to the division septum and controls the site specificity of cell division. Mol Microbiol 24:905–915. doi:10.1046/j.1365-2958.1997.3811764.x. 9219999

[24] Flärdh K. 2003. Essential role of DivIVA in polar growth and morphogenesis in *Streptomyces coelicolor* A3(2). Mol Microbiol 49:1523–1536. doi:10.1046/j.1365-2958.2003.03660.x. 12950918

[25] McCormick JR. 2009. Cell division is dispensable but not irrelevant in *Streptomyces*. Curr Opin Microbiol 12:689–698. doi:10.1016/j.mib.2009.10.004. 19889570

[26] Bagchi S, Tomenius H, Belova LM, Ausmees N. 2008. Intermediate filament-like proteins in bacteria and a cytoskeletal function in *Streptomyces*. Mol Microbiol 70:1037–1050. doi:10.1111/j.1365-2958.2008.06473.x. 18976278 PMC2680258

[27] Celler K, Koning RI, Koster AJ, van Wezel GP. 2013. Multidimensional view of the bacterial cytoskeleton. J Bacteriol 195:1627–1636. doi:10.1128/JB.02194-12. 23417493 PMC3624557

[28] Holmes NA, Walshaw J, Leggett RM, Thibessard A, Dalton KA, Gillespie MD, Hemmings AM, Gust B, Kelemen GH. 2013. Coiled-coil protein Scy is a key component of a multiprotein assembly controlling polarized growth in *Streptomyces*. Proc Natl Acad Sci U S A 110:E397–E406. doi:10.1073/pnas.1210657110. 23297235 PMC3562780

[29] Fuchino K, Bagchi S, Cantlay S, Sandblad L, Wu D, Bergman J, Kamali-Moghaddam M, Flärdh K, Ausmees N. 2013. Dynamic gradients of an intermediate filament-like cytoskeleton are recruited by a polarity landmark during apical growth. Proc Natl Acad Sci U S A 110:E1889–97. doi:10.1073/pnas.1305358110. 23641002 PMC3666699

[30] Girard G, Traag BA, Sangal V, Mascini N, Hoskisson PA, Goodfellow M, van Wezel GP. 2013. A novel taxonomic marker that discriminates between morphologically complex actinomycetes. Open Biol 3:130073. doi:10.1098/rsob.130073. 24153003 PMC3814722

[31] Girard G, Willemse J, Zhu H, Claessen D, Bukarasam K, Goodfellow M, van Wezel GP. 2014. Analysis of novel *kitasatosporae* reveals significant evolutionary changes in conserved developmental genes between *Kitasatospora* and *Streptomyces*. Antonie Van Leeuwenhoek 106:365–380. doi:10.1007/s10482-014-0209-1. 24958203

[32] Ramijan K, Ultee E, Willemse J, Zhang Z, Wondergem JAJ, van der Meij A, Heinrich D, Briegel A, van Wezel GP, Claessen D. 2018. Stress-induced formation of cell wall-deficient cells in filamentous actinomycetes. Nat Commun 9:5164. doi:10.1038/s41467-018-07560-9. 30514921 PMC6279842

[33] Claessen D, Errington J. 2019. Cell wall-deficiency as a coping strategy for stress. Trends Microbiol 27:1025–1033. doi:10.1016/j.tim.2019.07.008. 31420127

[34] Errington J, Mickiewicz K, Kawai Y, Wu LJ. 2016. L-form bacteria, chronic diseases and the origins of life. Philos Trans R Soc B 371:20150494. doi:10.1098/rstb.2015.0494. PMC5052740 27672147

[35] Leaver M, Dominguez-Cuevas P, Coxhead JM, Daniel RA, Errington J. 2009. Life without a wall or division machine in *Bacillus subtilis*. Nature 457:849–853. doi:10.1038/nature07742. 19212404

[36] Mercier R, Kawai Y, Errington J. 2016. Wall proficient E. coli capable of sustained growth in the absence of the Z-ring division machine. Nat Microbiol 1:16091. doi:10.1038/nmicrobiol.2016.91. 27573111

[37] Studer P, Staubli T, Wieser N, Wolf P, Schuppler M, Loessner MJ. 2016. Proliferation of *Listeria monocytogenes* L-form cells by formation of internal and external vesicles. Nat Commun 7:13631. doi:10.1038/ncomms13631. 27876798 PMC5123018

[38] Mercier R, Kawai Y, Errington J. 2013. Excess membrane synthesis drives a primitive mode of cell proliferation. Cell 152:997–1007. doi:10.1016/j.cell.2013.01.043. 23452849

[39] Errington J. 2013. L-form bacteria, cell walls and the origins of life. Open Biol 3:120143. doi:10.1098/rsob.120143. 23303308 PMC3603455

[40] Briers Y, Walde P, Schuppler M, Loessner MJ. 2012. How did bacterial ancestors reproduce? Lessons from L-form cells and giant lipid vesicles: multiplication similarities between lipid vesicles and L-form bacteria. Bioessays 34:1078–1084. doi:10.1002/bies.201200080. 23108858

[41] Cambré A, Zimmermann M, Sauer U, Vivijs B, Cenens W, Michiels CW, Aertsen A, Loessner MJ, Noben JP, Ayala JA, Lavigne R, Briers Y. 2015. Metabolite profiling and peptidoglycan analysis of transient cell wall-deficient bacteria in a new *Escherichia coli* model system. Environ Microbiol 17:1586–1599. doi:10.1111/1462-2920.12594. 25142185

[42] Mercier R, Kawai Y, Errington J. 2014. General principles for the formation and proliferation of a wall-free (L-form) state in bacteria. Elife 3:e04629. doi:10.7554/eLife.04629. PMC4244569 25358088

[43] Zacchetti B, Willemse J, Recter B, van Dissel D, van Wezel GP, Wösten HAB, Claessen D. 2016. Aggregation of germlings is a major contributing factor towards mycelial heterogeneity of *Streptomyces*. Sci Rep 6:27045. doi:10.1038/srep27045. 27244565 PMC4886682

[44] El-Gebali S, Mistry J, Bateman A, Eddy SR, Luciani A, Potter SC, Qureshi M, Richardson LJ, Salazar GA, Smart A, Sonnhammer ELL, Hirsh L, Paladin L, Piovesan D, Tosatto SCE, Finn RD. 2019. The Pfam protein families database in 2019. Nucleic Acids Res 47:D427–D432. doi:10.1093/nar/gky995. 30357350 PMC6324024

[45] Qi LS, Larson MH, Gilbert LA, Doudna JA, Weissman JS, Arkin AP, Lim WA. 2013. Repurposing CRISPR as an RNA-guided platform for sequence-specific control of gene expression. Cell 152:1173–1183. doi:10.1016/j.cell.2013.02.022. 23452860 PMC3664290

[46] Ultee E, van der Aart LT, Zhang L, van Dissel D, Diebolder CA, van Wezel GP, Claessen D, Briegel A. 2020. Teichoic acids anchor distinct cell wall lamellae in an apically growing bacterium. Commun Biol 3:314. doi:10.1038/s42003-020-1038-6. 32555532 PMC7300013

[47] McCormick JR, Su EP, Driks A, Losick R. 1994. Growth and viability of *Streptomyces coelicolor* mutant for the cell division gene *ftsZ*. Mol Microbiol 14:243–254. doi:10.1111/j.1365-2958.1994.tb01285.x. 7830569

[48] Bennett JA, Aimino RM, McCormick JR. 2007. *Streptomyces coelicolor* genes *ftsL* and *divIC* play a role in cell division but are dispensable for colony formation. J Bacteriol 189:8982–8992. doi:10.1128/JB.01303-07. 17951394 PMC2168613

[49] Bennett JA, Yarnall J, Cadwallader AB, Kuennen R, Bidey P, Stadelmaier B, McCormick JR. 2009. Medium-dependent phenotypes of *Streptomyces coelicolor* with mutations in *ftsI* or *ftsW*. J Bacteriol 191:661–664. doi:10.1128/JB.01048-08. 18978049 PMC2620802

[50] Mistry BV, Del Sol R, Wright C, Findlay K, Dyson P. 2008. FtsW is a dispensable cell division protein required for Z-ring stabilization during sporulation septation in *Streptomyces coelicolor*. J Bacteriol 190:5555–5566. doi:10.1128/JB.00398-08. 18556789 PMC2519378

[51] Labeda DP, Goodfellow M, Brown R, Ward AC, Lanoot B, Vanncanneyt M, Swings J, Kim SB, Liu Z, Chun J, Tamura T, Oguchi A, Kikuchi T, Kikuchi H, Nishii T, Tsuji K, Yamaguchi Y, Tase A, Takahashi M, Sakane T, Suzuki KI, Hatano K. 2012. Phylogenetic study of the species within the family *Streptomycetaceae*. Antonie Van Leeuwenhoek 101:73–104. doi:10.1007/s10482-011-9656-0. 22045019

[52] Stuttard C. 1982. Temperate phages of *Streptomyces venezuelae*: lysogeny and host specificity shown by phages SV1 and SV2. J Gen Microbiol 128:115–121. doi:10.1099/00221287-128-1-115.

[53] Sun J, Kelemen GH, Fernández-Abalos JM, Bibb MJ. 1999. Green fluorescent protein as a reporter for spatial and temporal gene expression in *Streptomyces coelicolor* A3(2). Microbiology 145:2221–2227. doi:10.1099/00221287-145-9-2221. 10517575

[54] Gust B, Challis GL, Fowler K, Kieser T, Chater KF. 2003. PCR-targeted *Streptomyces* gene replacement identifies a protein domain needed for biosynthesis of the sesquiterpene soil odor geosmin. Proc Natl Acad Sci U S A 100:1541–1546. doi:10.1073/pnas.0337542100. 12563033 PMC149868

[55] Ramijan K, van Wezel GP, Claessen D. 2017. Genome sequence of the filamentous actinomycete *Kitasatospora viridifaciens*. Genome Announc 5:e01560-16. doi:10.1128/genomeA.01560-16. 28183757 PMC5331497

[56] Vara J, Lewandowska-Skarbek M, Wang YG, Donadio S, Hutchinson CR. 1989. Cloning of genes governing the deoxysugar portion of the erythromycin biosynthesis pathway in S*accharopolyspora erythraea* (*Streptomyces erythreus*). J Bacteriol 171:5872–5881. doi:10.1128/jb.171.11.5872-5881.1989. 2681144 PMC210448

[57] Świątek MA, Tenconi E, Rigali S, van Wezel GP. 2012. Functional analysis of the N-acetylglucosamine metabolic genes of *Streptomyces coelicolor* and role in control of development and antibiotic production. J Bacteriol 194:1136–1144. doi:10.1128/JB.06370-11. 22194457 PMC3294797

[58] Motamedi H, Shafiee A, Cai S-J. 1995. Integrative vectors for heterologous gene expression in *Streptomyces* spp. Gene 160:25–31. doi:10.1016/0378-1119(95)00191-8. 7628712

[59] Gregory MA, Till R, Smith MCM. 2003. Integration site for *Streptomyces* phage phiBT1 and development of site-specific integrating vectors. J Bacteriol 185:5320–5323. doi:10.1128/jb.185.17.5320-5323.2003. 12923110 PMC180994

[60] Kieser T, Bibb MJ, Buttner MJ, Chater KF, Hopwood DA. 2000. Practical Streptomyces genetics. The John Innes Foundation, Norwich, United Kingdom.

[61] van der Aart LT, Lemmens N, van Wamel WJ, van Wezel GP. 2016. Substrate inhibition of VanA by d-alanine reduces vancomycin resistance in a VanX-dependent manner. Antimicrob Agents Chemother 60:4930–4939. doi:10.1128/AAC.00276-16. 27270282 PMC4958238

[62] van der Aart LT, Spijksma GK, Harms A, Vollmer W, Hankemeier T, van Wezel GP. 2018. High-resolution analysis of the peptidoglycan composition in *Streptomyces coelicolor*. J Bacteriol 200:e00290-18. doi:10.1128/JB.00290-18. 30061355 PMC6153666

[63] Kühner D, Stahl M, Demircioglu DD, Bertsche U. 2014. From cells to muropeptide structures in 24 h: peptidoglycan mapping by UPLC-MS. Sci Rep 4:7494. doi:10.1038/srep07494. 25510564 PMC4267204

[64] Medema MH, Takano E, Breitling R. 2013. Detecting sequence homology at the gene cluster level with MultiGeneBlast. Mol Biol Evol 30:1218–1223. doi:10.1093/molbev/mst025. 23412913 PMC3670737

[65] Zhu H, Swierstra J, Wu C, Girard G, Choi YH, van Wamel W, Sandiford SK, van Wezel GP. 2014. Eliciting antibiotics active against the ESKAPE pathogens in a collection of actinomycetes isolated from mountain soils. Microbiology (Reading) 160:1714–1725. doi:10.1099/mic.0.078295-0. 24794971

[66] Finn RD, Clements J, Eddy SR. 2011. HMMER web server: interactive sequence similarity searching. Nucleic Acids Res 39:W29–W37. doi:10.1093/nar/gkr367. 21593126 PMC3125773

[67] Katoh K, Standley DM. 2013. MAFFT multiple sequence alignment software version 7: improvements in performance and usability. Mol Biol Evol 30:772–780. doi:10.1093/molbev/mst010. 23329690 PMC3603318

[68] Shen W, Le S, Li Y, Hu F. 2016. SeqKit: a cross-platform and ultrafast toolkit for FASTA/Q file manipulation. PLoS One 11:e0163962. doi:10.1371/journal.pone.0163962. 27706213 PMC5051824

[69] Stamatakis A. 2014. RAxML version 8: a tool for phylogenetic analysis and post-analysis of large phylogenies. Bioinformatics 30:1312–1313. doi:10.1093/bioinformatics/btu033. 24451623 PMC3998144

[70] Letunic I, Bork P. 2019. Interactive Tree Of Life (iTOL) v4: recent updates and new developments. Nucleic Acids Res 47 (W1):W256–W259. doi:10.1093/nar/gkz239. 30931475 PMC6602468

[71] Eddy SR. 2011. Accelerated profile HMM searches. PLoS Comput Biol 7:e1002195. doi:10.1371/journal.pcbi.1002195. 22039361 PMC3197634

[72] Shannon P, Markiel A, Ozier O, Baliga NS, Wang JT, Ramage D, Amin N, Schwikowski B, Ideker T. 2003. Cytoscape: a software environment for integrated models of biomolecular interaction networks. Genome Res 13:2498–2504. doi:10.1101/gr.1239303. 14597658 PMC403769

[73] Hanahan D. 1983. Studies on transformation of *Escherichia coli* with plasmids. J Mol Biol 166:557–580. doi:10.1016/s0022-2836(83)80284-8. 6345791

[74] Yanisch-Perron C, Vieira J, Messing J. 1985. Improved M13 phage cloning vectors and host strains: nucleotide sequences of the M13mp18 and pUC19 vectors. Gene 33:103–119. doi:10.1016/0378-1119(85)90120-9. 2985470

[75] MacNeil DJ, Gewain KM, Ruby CL, Dezeny G, Gibbons PH, MacNeil T. 1992. Analysis of *Streptomyces avermitilis* genes required for avermectin biosynthesis utilizing a novel integration vector. Gene 111:61–68. doi:10.1016/0378-1119(92)90603-m. 1547955

[76] Jerpseth B, Kretz BL. 1993. SCS110: *dam-, dcm-, endA-* Epicurian coli competent cells. Strategies 6:22.

[77] Bierman M, Logan R, O'Brien K, Seno ET, Rao RN, Schoner BE. 1992. Plasmid cloning vectors for the conjugal transfer of DNA from *Escherichia coli* to *Streptomyces* spp. Gene 116:43–49. doi:10.1016/0378-1119(92)90627-2. 1628843

[78] Bibb MR, Janssen GR, Ward JM. 1985. Cloning and analysis of the promoter region of the erythromycin resistance gene (*ermE*) of *Streptomyces erythraeus*. Gene 38(1–3):215–226. doi:10.1016/0378-1119(85)90220-3. 2998943

