# Supplementary figures and images for: An Alternative and Conserved Cell Wall Enzyme That Can Substitute for the Lipid II Synthase MurG
Source: mBio. 2021 Apr 6;12(2):e03381-20. doi: 10.1128/mBio.03381-20 (PMC8092295; doi:10.1128/mBio.03381-20)

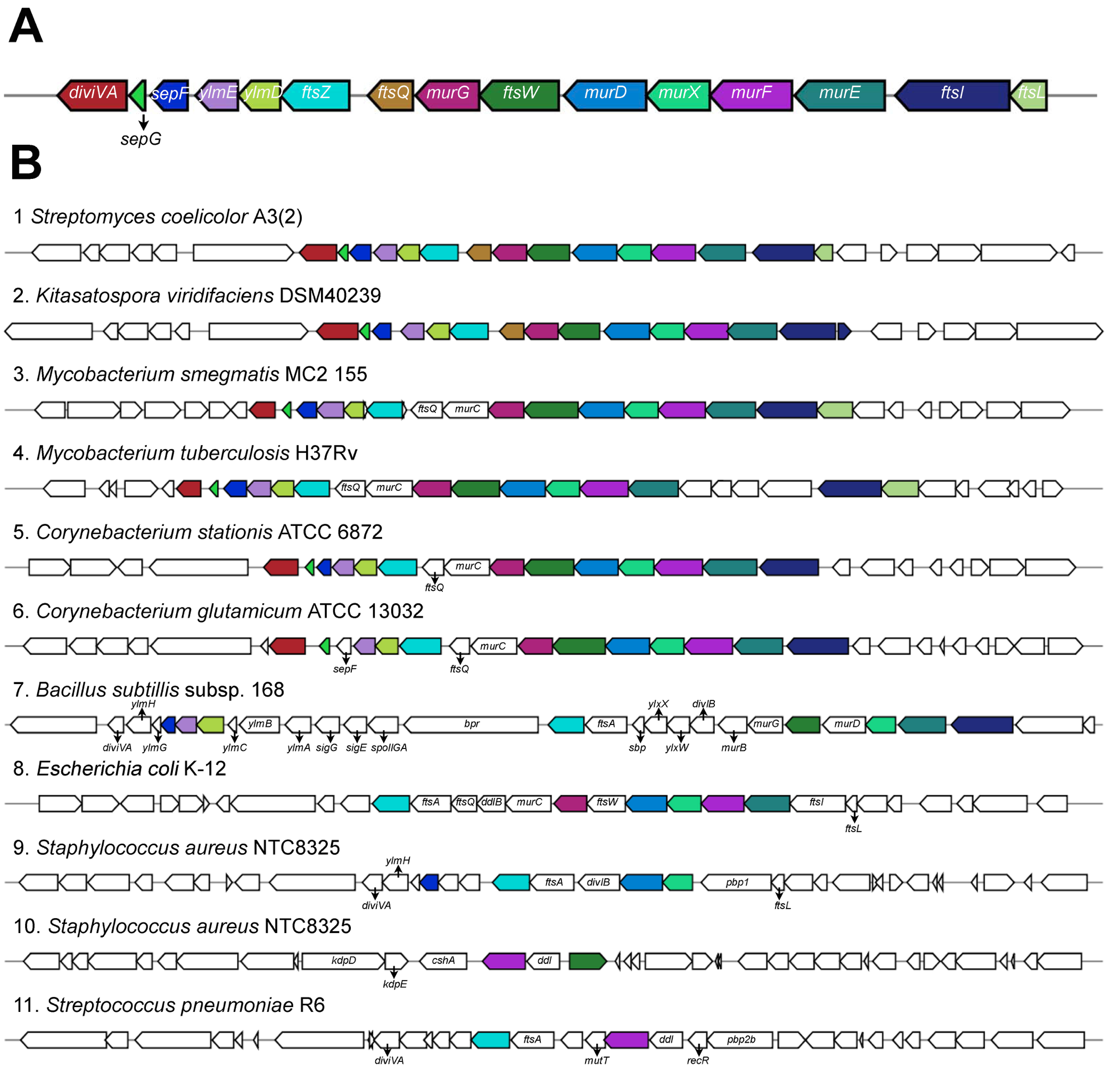

Supplement: FIG S2 [file mBio.03381-20-sf002.tif]

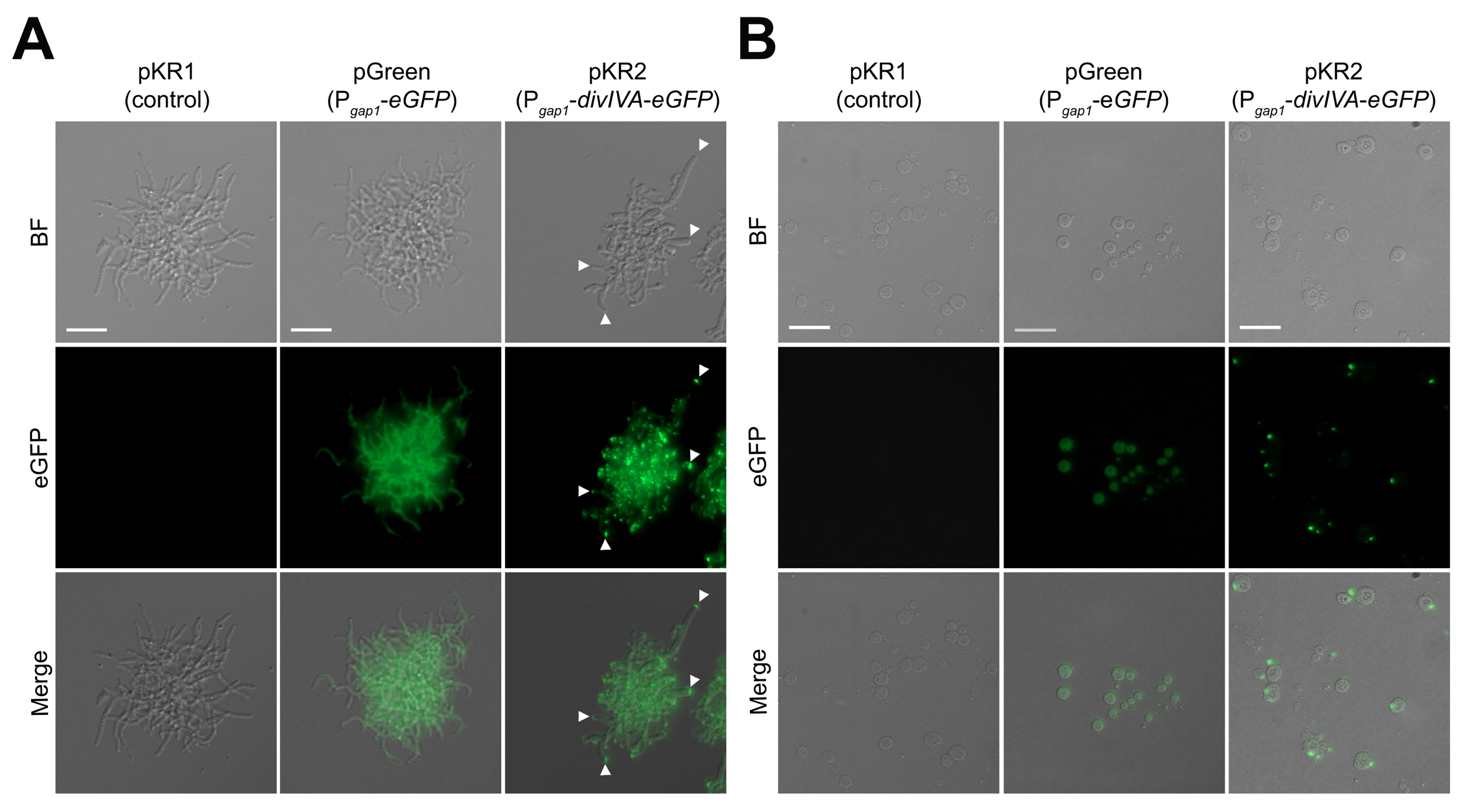

Supplement: FIG S3 [file mBio.03381-20-sf003.tif]

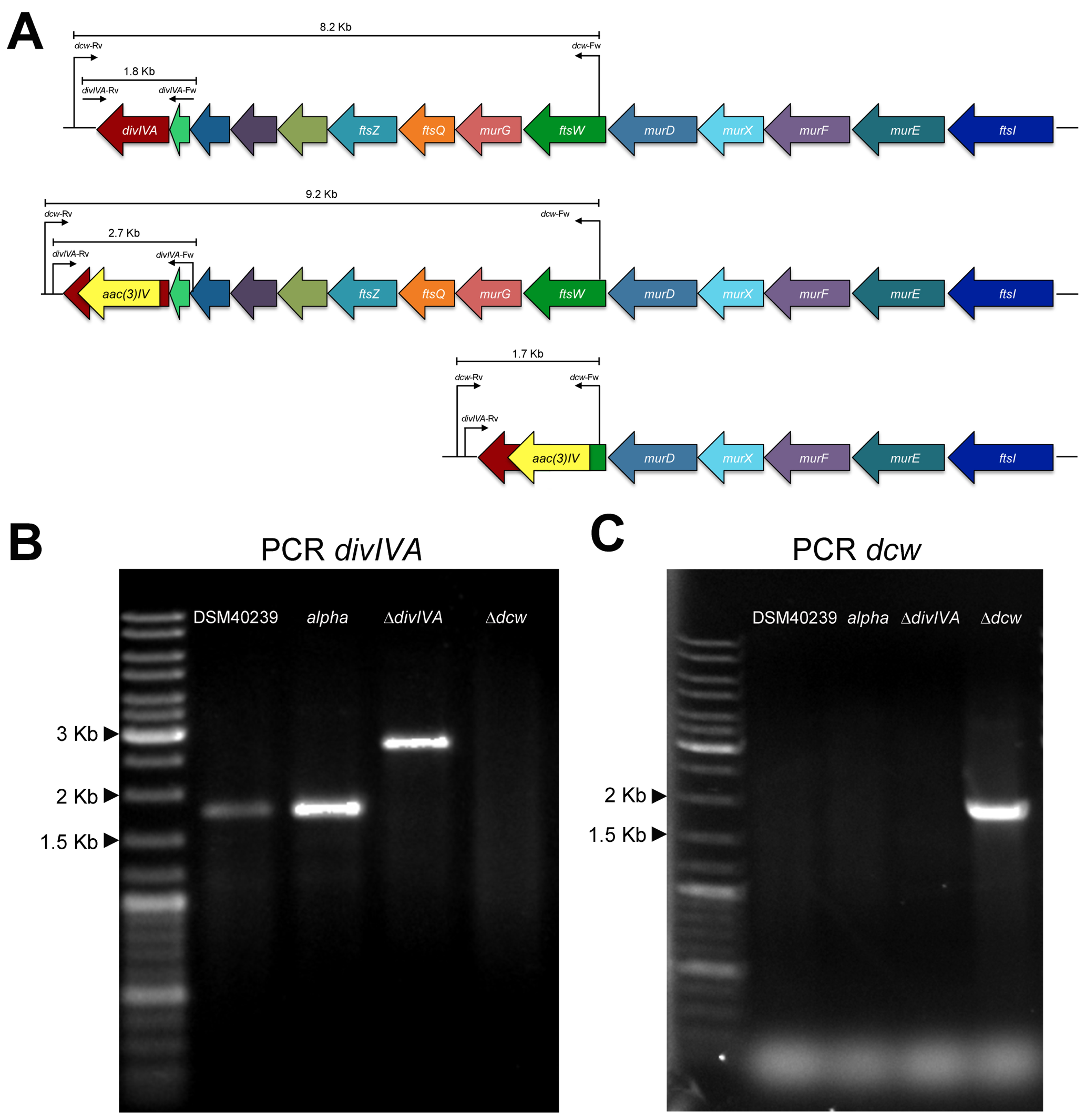

Supplement: FIG S4 [file mBio.03381-20-sf004.tif]

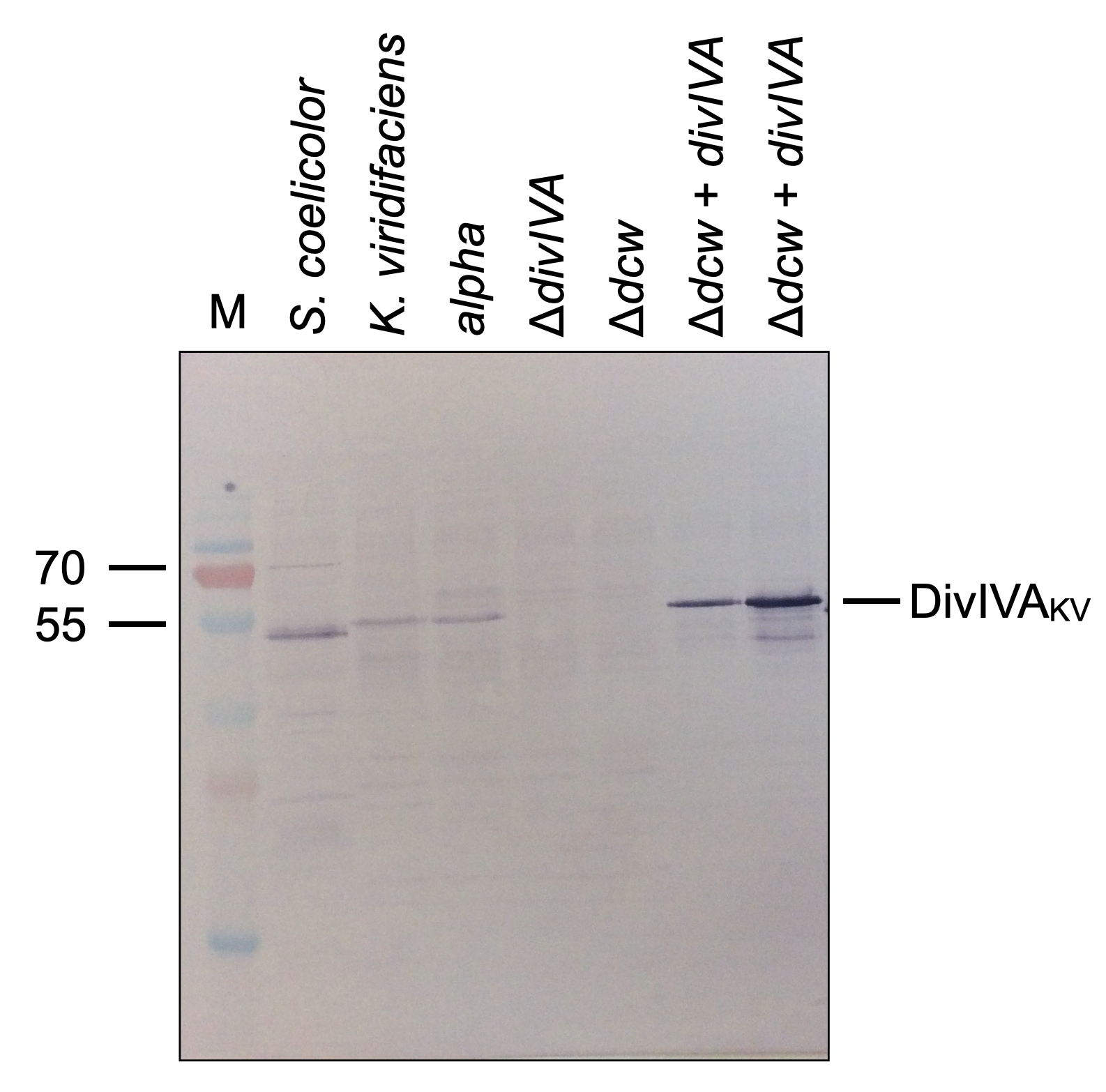

Supplement: FIG S5 [file mBio.03381-20-sf005.tif]

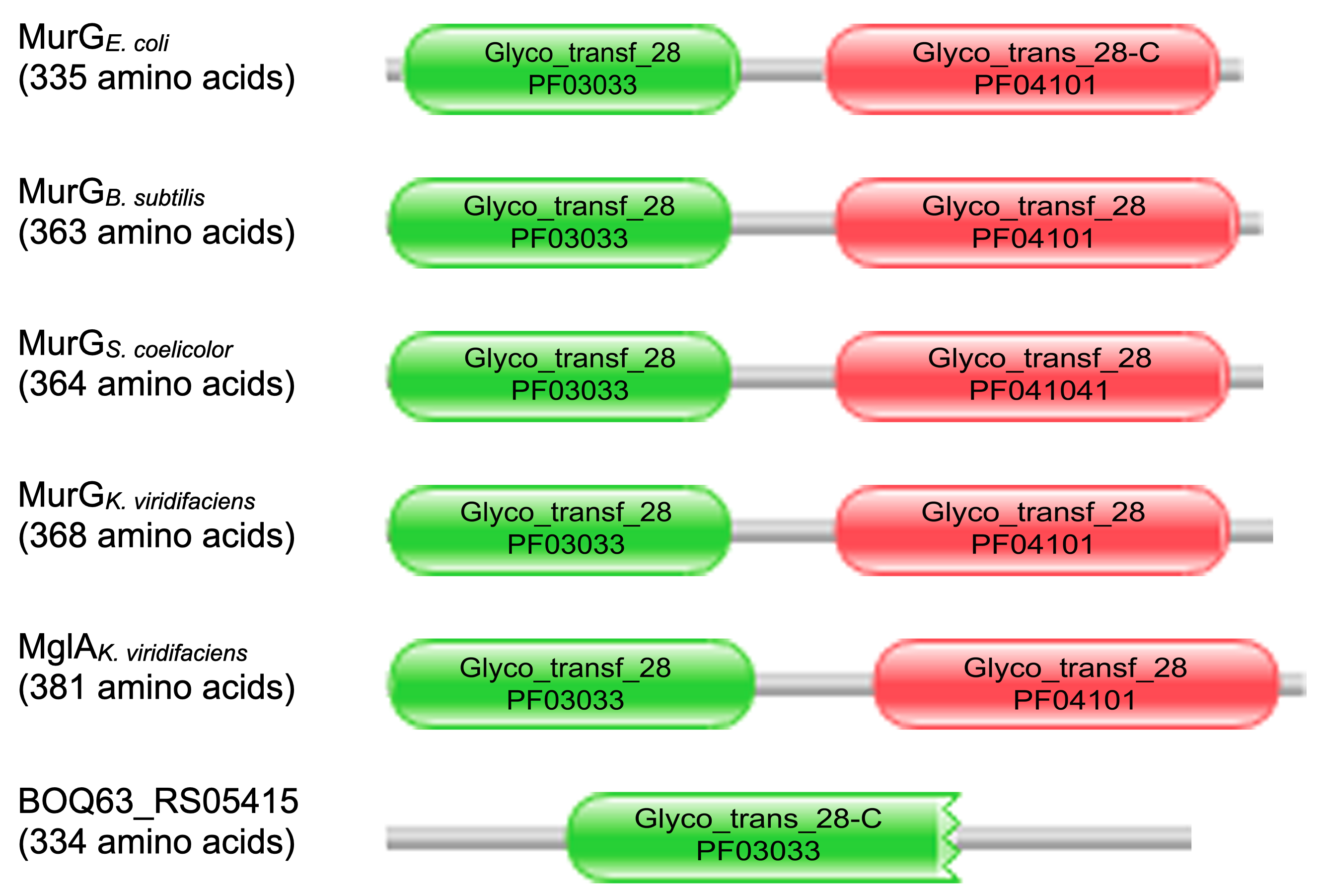

Supplement: FIG S6 [file mBio.03381-20-sf006.tif]

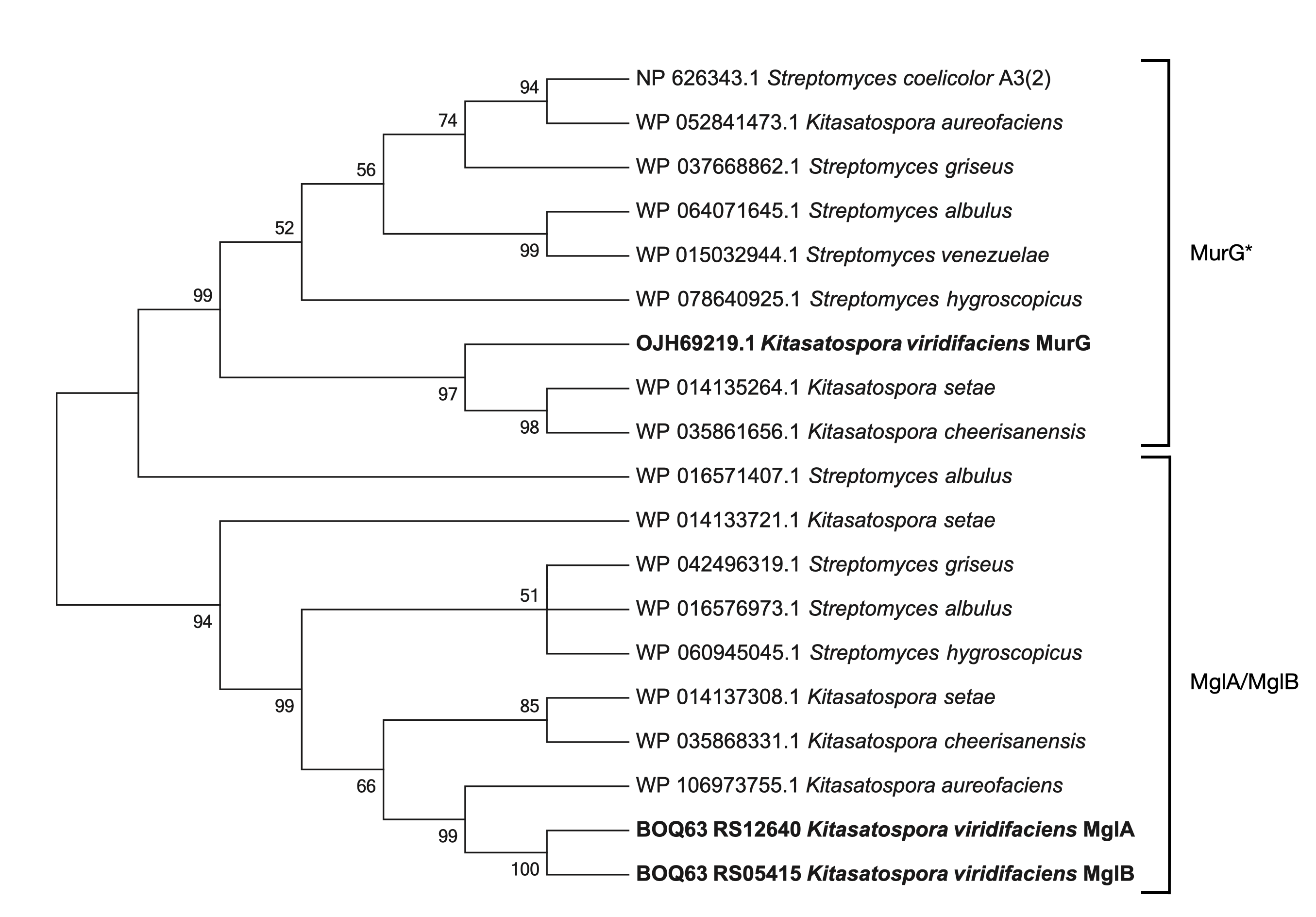

Supplement: FIG S7 [file mBio.03381-20-sf007.tif]

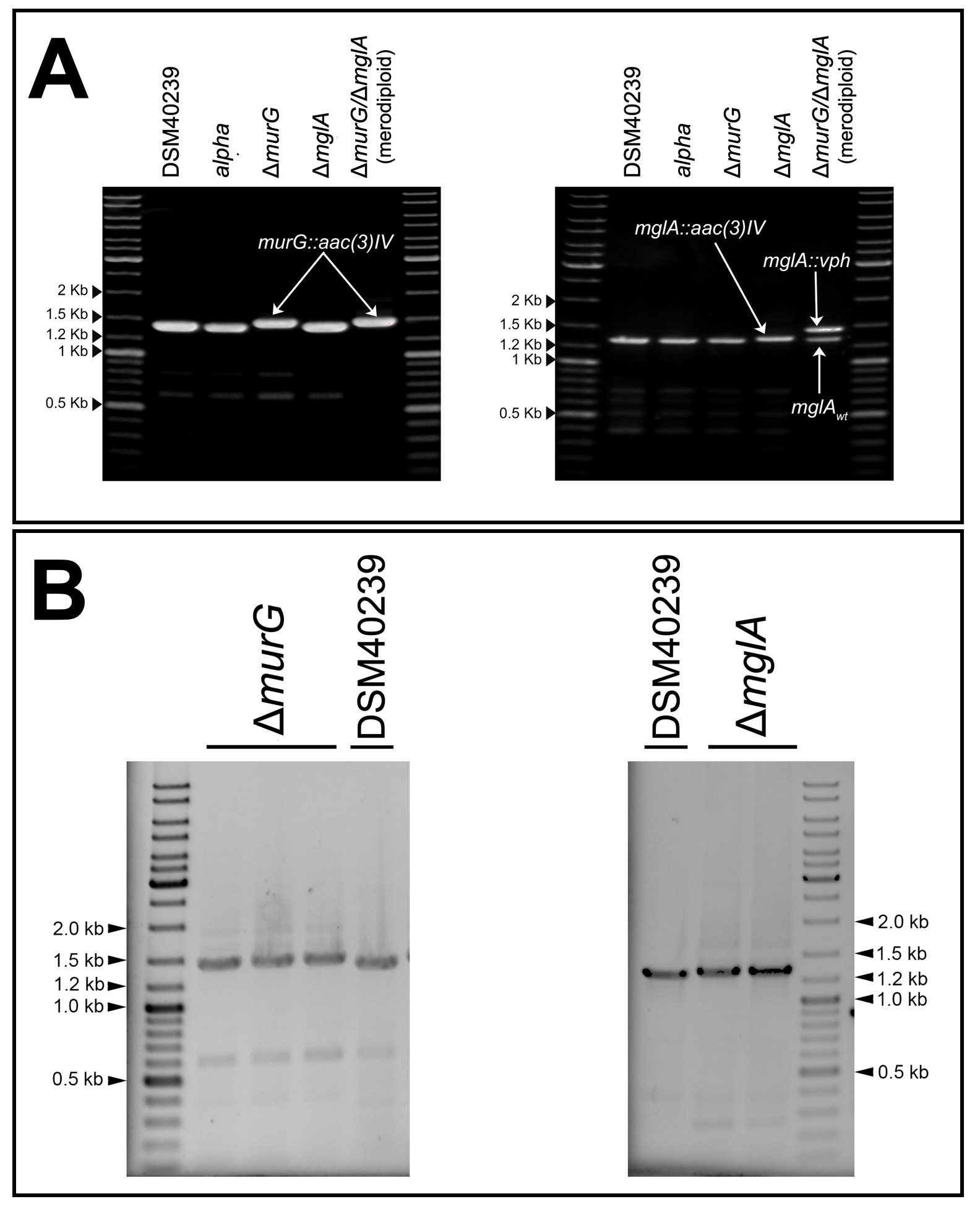

Supplement: FIG S8 [file mBio.03381-20-sf008.tif]
